# *HOTTIP*-Variants in Liver Cancer Metastasis Prognosis: A Clinical Study with Bioinformatics and siRNAs Targeting HOTTIP-WDR5 via Molecular Docking, a Step-Toward ncRNA Precision

**DOI:** 10.3390/ijms27052108

**Published:** 2026-02-24

**Authors:** Mona G. El-Sisi, Sara M. Radwan, Sameh S. Ali, Mohamed Y. Mostafa, Nadia M. Hamdy

**Affiliations:** 1 Department of Biochemistry and Molecular Biology, Faculty of Pharmacy, Ain Shams University, Cairo 11566, Egypt; 2 Research Department, Children’s Cancer Hospital Egypt-57357, Cairo 11562, Egypt; 3 Department of Clinical Oncology, Faculty of Medicine, Ain Shams University, Cairo 11566, Egypt

**Keywords:** HOTTIP, HCC, SNPs, haplotype, metastasis, survival, prognosis, diagnosis, molecular docking, siRNA, bioinformatics

## Abstract

Early detection of hepatocellular carcinoma (HCC) remains challenging due to limitations including the lack of reliable biomarkers. While molecular diagnostics hold promise, their use is limited because tissue biopsies are not routinely performed in HCC. Long non-coding RNAs (lncRNA), such as HOXA transcript at the distal tip (HOTTIP), have been implicated in HCC, with single-nucleotide polymorphisms forming haplotypes that may influence disease progression. This study investigated the clinical relevance of HOTTIP SNPs rs17501292 and rs2067087 in 198 Egyptian HCC patients (129 non-metastatic, 69 metastatic). Moreover, molecular docking was used to design small interfering RNAs (siRNAs) targeting HOTTIP. Genotypes *TT* and *TG* (rs17501292) and *GG* and *GC* (rs2067087) were associated with reduced metastatic risk compared to *GG* and *CC* genotypes, respectively. Survival analysis revealed that *TT* (rs17501292) and *GC* (rs2067087) genotypes correlated with improved outcomes. ROC curve analysis confirmed the diagnostic and prognostic value of specific genetic models, affirming their value as biomarkers for metastasis and survival. Molecular docking identified two promising therapeutic candidates. Overall, we can conclude that HOTTIP SNPs may serve as promising potential non-invasive biomarkers for HCC metastasis and prognosis, while the identified siRNAs offer a novel targeted therapeutic approach.

## 1. Introduction

Hepatocellular carcinoma (HCC) is a leading cause of cancer-related mortality and morbidity [1]. HCC is a multifactorial disease often triggered by hepatitis B or C viral infections, drug abuse, insulin resistance, or exposure to carcinogens [2,3]. Unfortunately, treatment options for HCC remain limited and are largely effective only during the early stage. At advanced stages, the prognosis is poor because of frequent metastasis, tumor recurrence, and the absence of curative therapies [4,5].

A notable hallmark of cancer is epigenetic alteration [6], particularly the involvement of long noncoding RNAs (lncRNAs), which are implicated in various diseases, including cancer, namely, liver, breast, colon, and lung cancers [7,8,9]. LncRNAs, which are over 200 nucleotides in length, play significant roles in cell proliferation, differentiation, and metabolism [8]. LncRNAs are often dysregulated in cancers and are key regulators of posttranscriptional processes [10,11]. Given their critical involvement in regulating multiple stages of gene expression, lncRNAs represent a novel area of molecular, bioinformatics, and clinical research [12].

Several lncRNAs, such as the HOXA transcript at the distal tip (HOTTIP), are associated with HCC progression and metastasis [13]. HOTTIP is a 3764-nucleotide-long spliced, polyadenylated noncoding RNA located at the 5′ terminal region of the HOXA locus, where it activates numerous 5′ HOXA genes [14]. HOTTIP has been associated with the pathophysiology of both communicable and noncommunicable diseases, including diabetes mellitus [15], Hirschsprung disease [16], and preeclampsia [17]. Studies have also linked *HOTTIP* expression to tumor stage, distant metastasis, lymph node metastasis (LNM), and overall survival (OS) in various malignancies [18]. Dysregulation of HOTTIP has been observed in multiple cancers, including HCC, where it impacts patient prognosis and survival [14,19]. HOTTIP has been reported to promote HCC cell proliferation and metastasis, suggesting its role as an oncogenic lncRNA in HCC [20]; if this role is related to genetic variations in this lncRNA, a hypothesis to be examined.

One of the interesting downstream pathways of HOTTIP is its interaction with WD Repeat-Containing Protein 5 (WDR5 protein) [21]. HOTTIP interacts directly with the WDR5 protein, facilitating the recruitment of WDR5/Mixed Lineage Leukemia (MLL) complexes to the HOXA locus, which in turn promotes Histone H3 lysine 4 (H3K4) methylation and activates HOXA gene transcription [21].

Genetic variations such as single-nucleotide polymorphisms (SNPs) are related to disease pathogenesis [22,23]. Moreover, SNPs often occur in clusters due to high linkage disequilibrium (LD) [24,25]. This leads to the shared inheritance of specific SNP groups known as haplotypes [26]. Haplotypes can influence the expression of coding and non-coding genes, including lncRNAs [27]. However, only a limited number of studies have explored SNPs within the oncogenic lncRNA *HOTTIP* gene (Gene ID: 100316868) [28,29,30]. Wang et al. identified specific HOTTIP SNPs as potential biomarkers for HCC risk and prognosis. Specifically, the haplotype “rs17501292-rs2067087-rs17427960” was significantly associated with a 1.91-fold increased risk of HCC in Chinese patient samples [30]. However, these SNPs and this haplotype have not yet been investigated in metastatic vs. non-metastatic HCC patients and have not been examined in Egyptian HCC patients.

Therefore, this study aimed to investigate the association of HOTTIP gene polymorphisms (rs17501292 and rs2067087) with metastasis, prognosis, and survival in Egyptian patients with hepatocellular carcinoma. We evaluated minor allele frequencies and genotype distributions in metastatic and non-metastatic HCC, assessed the association of these variants with metastatic risk, and examined their prognostic significance using survival analysis. In addition, the diagnostic and prognostic value of HOTTIP SNPs was explored using ROC and haplotype analyses, while molecular docking was employed to investigate potential siRNA strategies targeting HOTTIP-WDR5 interactions, providing mechanistic insights into HCC metastasis and future therapeutic directions.

## 2. Results

**Figure 1:**
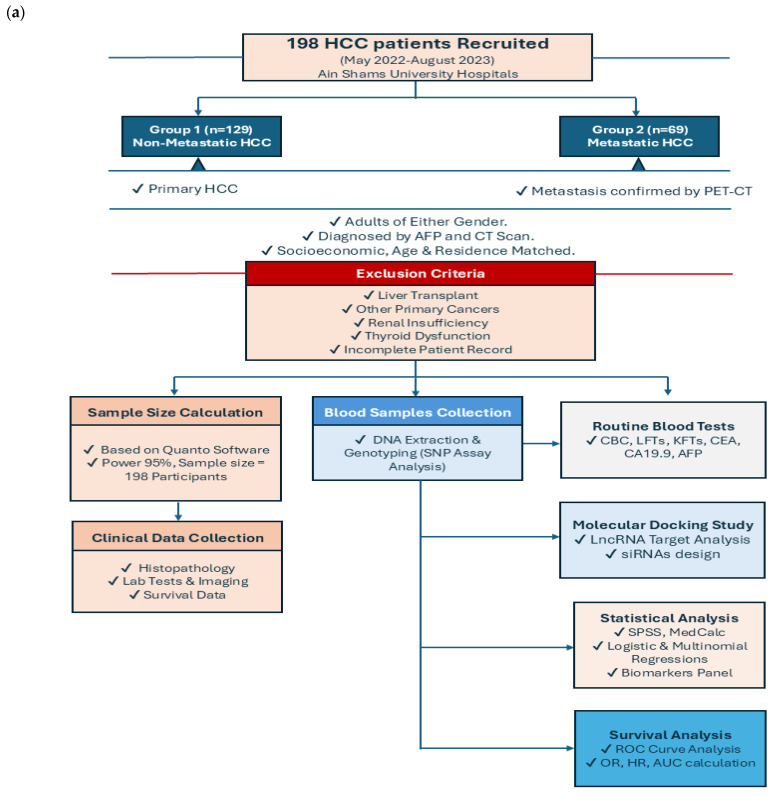

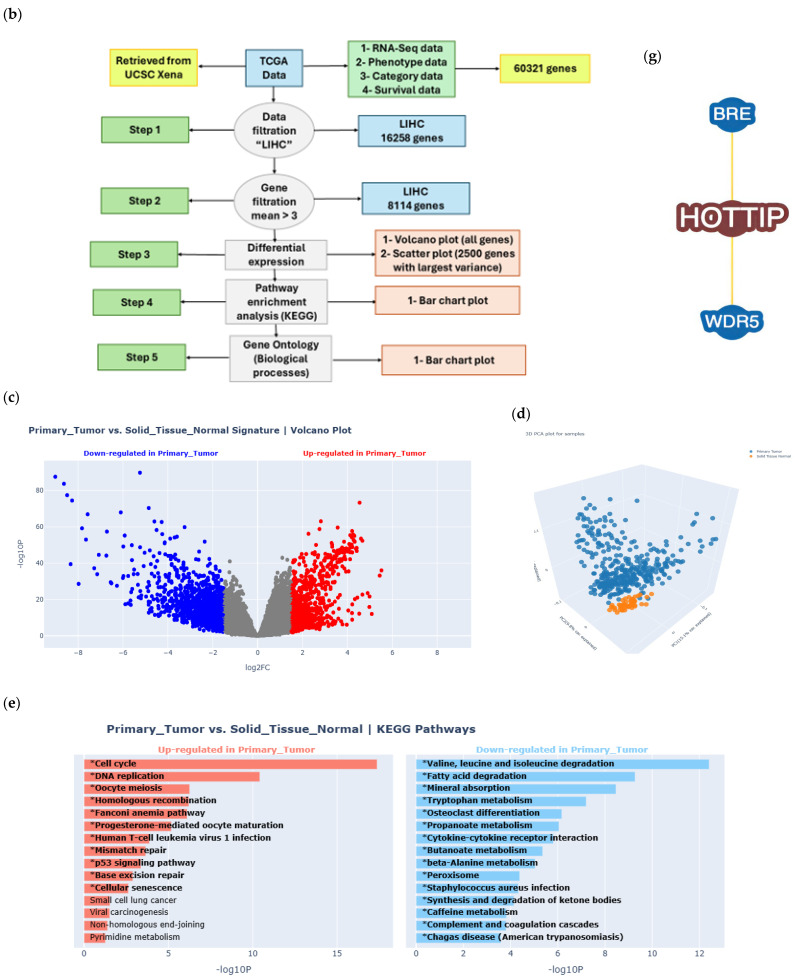

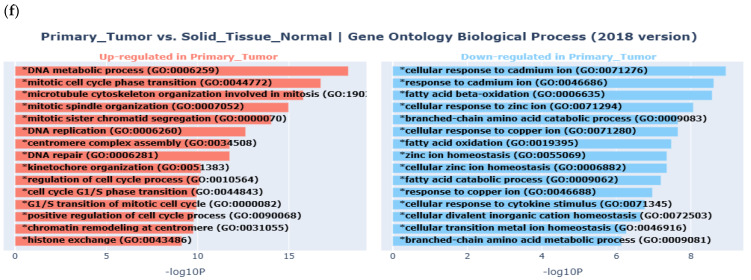
(**a**) Schematic flow chart of patient recruitment, selection criteria, and methodological steps of the study, including group allocation, sample size calculation, clinical and molecular analyses, and statistical evaluation (created by PowerPoint). (**b**) Data processing methodology (created by PowerPoint). (**c**) Volcano plot for LIHC primary_tumor vs. solid_tissue_normal upregulated and downregulated genes. The figure contains an interactive scatter plot that displays the log2-fold changes and statistical significance of each gene calculated via DGE analysis. Upregulated genes with log2FC > 1.5 and *p* value < 0.05 are shown in red, and downregulated genes with log2FC < −1.5 and *p* value < 0.05 are shown in blue. (**d**) 3D PCA plot for LIHC samples retrieved from online datasets using 2500 genes with the largest variance. The figure displays an interactive, three-dimensional scatter plot of the data. Each point represents a gene expression sample. Samples with similar gene expression profiles are closer in three-dimensional space. (**e**) Enrichment analysis results for the primary_tumor and solid_tissue_normal pathways of LIHC. The figures contain interactive bar charts displaying the results of the Gene Ontology enrichment analysis generated via Enrichr. The x-axis indicates the −log10(*p*-value) for each term. Significant terms are highlighted in bold. (**f**) GO Enrichment Analysis results for primary_tumor vs. solid_tissue_normal. The figures contain interactive bar charts displaying the results of the Gene Ontology enrichment analysis generated via Enrichr. The x-axis indicates the −log10(*p* value) for each term. Significant terms are highlighted in bold. (**g**) HOTTIP-WDR5 interaction network generated via BioGRID^4.4^ (https://downloads.thebiogrid.org/BioGRID/Release-Archive/BIOGRID-4.4.202/, accessed on 21 April 2025).

A flowchart summarizing the methodology and patients’ selection criteria can be found in (Figure 1a).

### 2.1. Bioinformatics Results

The data analysis flow chart is shown in (Figure 1b).

#### 2.1.1. Differential Gene Expression (DGE) Results in Liver Cancer

Gene expression signatures of changes in gene expression patterns that arise from cellular disturbances, such as drug treatments, gene knockdowns, or diseases, measured via DGE methods as volcano plots are shown in (Figure 1c), where upregulated genes in Liver Hepatocellular Carcinoma (LIHC), including all *HOXA* genes, are presented in red and downregulated genes in LIHC are presented in blue. Moreover, the expressed genes in LIHC, along with their Log2FC, average expression, and *p*-value are attached as a supplementary Excel file (Supplementary Table S1).

#### 2.1.2. Principal Component Analysis (PCA) Results

Converting gene expression values into Principal Components (PCs), a set of linearly uncorrelated variables that capture the most significant variance in the dataset is visualized via a scatter plot to illustrate sample relationships. The 3D plot for PCA is shown in (Figure 1d).

#### 2.1.3. Enrichment Analysis Results

The results of the pathway enrichment analysis are shown in (Figure 1e), where the contributing pathways in LIHC pathogenesis are arranged in descending order, with upregulated pathways shown in red and downregulated pathways shown in blue. The results of the Gene Ontology (GO) enrichment analysis are shown in (Figure 1f), where the contributing biological processes in LIHC pathogenesis are arranged in descending order, while upregulated biological processes are shown in red, and downregulated biological processes are shown in blue.

#### 2.1.4. Protein–Protein Interaction (PPI) and Gene–Protein Interaction

WD Repeat Domain 5 protein, the major downstream target of HOTTIP, was retrieved from the UniProt database via the accession number P61964 as well as the BioGRID^4.4^ database (https://downloads.thebiogrid.org/BioGRID/Release-Archive/BIOGRID-4.4.202/, accessed on 21 April 2025) (Figure 1g) presents the HOTTIP–WDR5 interaction network.

#### 2.1.5. Selected Polymorphism Sites in the lncRNA HOTTIP

**Figure 2:**
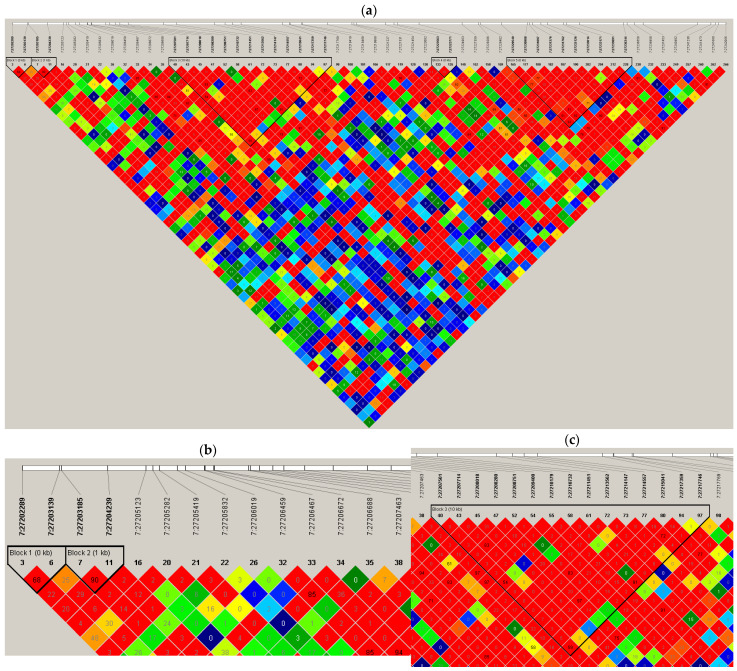
Linkage disequilibrium (LD) heatmap of single-nucleotide polymorphisms (SNPs) across Chromosome 7: (**a**). The zoomed-out map. (**b**). After zooming in for more clarification. (**c**). After zooming in from another area for more clarification, and for the choice of SNPs. Bright Red represents the strongest association, meaning the markers are almost always inherited together; Shades of Pink or Light Red indicate a strong statistical significance but with a lower LD value, suggesting that while the linkage is real, some historical recombination has occurred; Blue is used for “coincident” linkage often due to a small sample size or a very low allele frequency; White indicates a complete lack of evidence for linkage, signifying that the markers are likely far apart or in a region of high recombination.

To select polymorphic sites, the resulting HapMap (Figure 2) shows Pairwise LD between SNPs in the analyzed genomic region. Each square corresponds to a comparison between two SNPs, with the color indicating the degree of LD as measured by r^2^. The r^2^ metric ranges from 0 (no LD) to 1 (complete LD), with higher values indicating stronger correlations between alleles at two loci. The color gradient reflects the LD strength: red denotes high LD (r^2^ > 0.8), orange/yellow denotes moderate LD (r^2^ ≈ 0.4–0.8), and green/blue denotes low LD (r^2^ < 0.4).

After block identification, many SNPs were detected, among which 2 SNPs with high LD (>0.8) were selected: rs17501292-rs2067087 [30,31].

### 2.2. Clinical, Demographic, and Laboratory Characteristics of the Studied Groups

The demographic, laboratory, and pathological features of the patients in the studied groups are shown in (Table 1). There was no statistically significant difference, with *p*  >  0.05, between the metastatic and non-metastatic groups in terms of age, which indicates proper matching between the studied groups.

**Figure 3:**
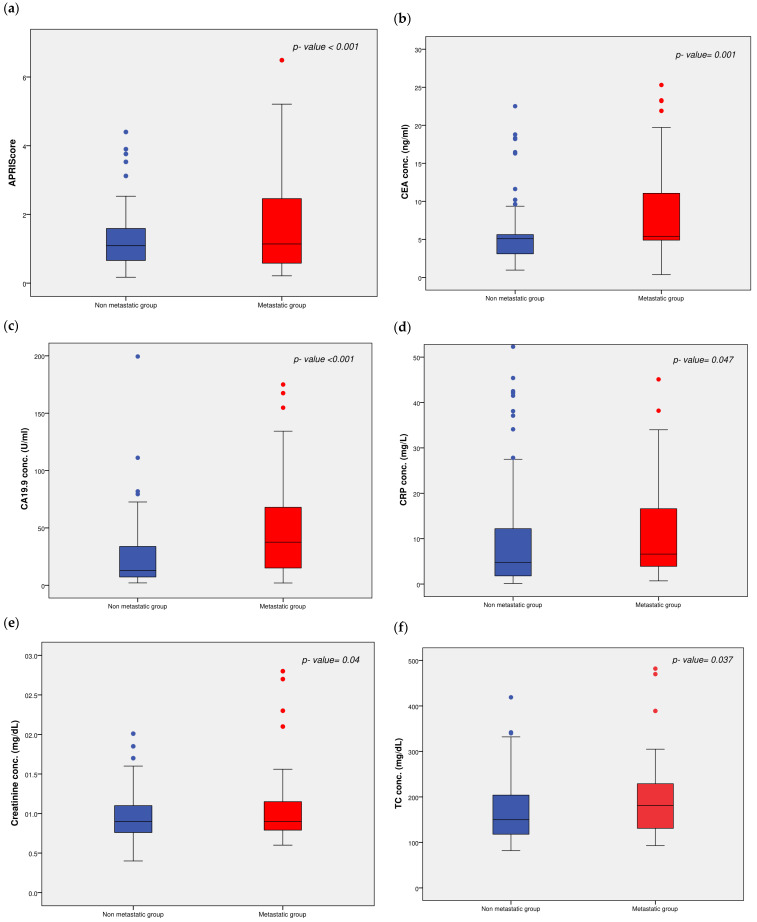
Patient groups comparison with respect to (**a**) APRI score. (**b**) CEA. (**c**) CA19.9. (**d**) CRP. (**e**) Creatinine. (**f**) TC. The concentrations of different variables are presented in box plots (red for metastatic group and blue for the non-metastatic). The maximum and minimum values are represented by bars; the non-metastatic group (n = 129), the metastatic group (n = 69); the dots represent outlier samples (red for metastatic group and blue for the non-metastatic). [APRI score, AST-to-Platelet Ratio Index; CEA, Carcinoembryonic Antigen; CA19.9, Cancer Antigen 19.9; CRP, C-Reactive Protein; TC, Total Cholesterol].

As shown, there was a significant difference in some anthropometric and biochemical measurements between the study groups. CRP, creatinine, the platelet count, the ALT-to-platelet ratio index (APRI) score, CEA, CA19.9, TC, EO, and RBCs were significantly greater, whereas the MCV, MCH, and MCHC were lower in the metastatic group than in the non-metastatic group. Box plots showing the differences between the two groups with respect to some biochemical measurements are shown in (Figure 3a–f).

Moreover, the clinical characteristics of the U.S. patients are shown in (Table 2).

With respect to ultrasonography, 51.9% of non-metastatic patients had lesions in the right focal lobe, 13.2% had lesions in the left focal lobe, 33.3% had lesions in the caudate lobe, and 1.6% had focal lesions in both lobes. On the other hand, in the metastatic group, 60.9% of the patients had lesions in the right focal lobe, 8.7% had lesions in the left focal lobe, and 1.4% had lesions in the caudate lobe, whereas 29% had focal lesions in both lobes. Approximately 59% of non-metastatic HCC patients had thrombosis in the portal vein, whereas 50.7% of metastatic HCC patients had thrombosis in the portal vein. Abdominal lymph nodes were observed in only 38.8% of non-metastatic HCC patients, whereas they were observed in 55.1% of patients in the metastatic group. In the metastatic group, the metastatic site distributions were as follows: kidney (26.1%), far/distant LN (8.7%), lung (27.6%), bone (20.3%), colon (10.1%), and prostate (7.2%) (Table 2). The adjusted *p* values for the effects of the covariates of both age and gender, performed by General Linear Model (GLM), are presented in (Table 2).

### 2.3. Biomarkers Panel Performance

**Figure 4:**
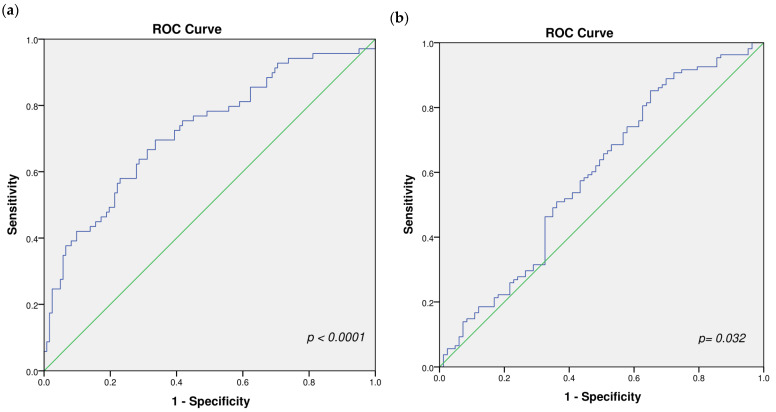
ROC curve analysis of the selected panel performance in discriminating (**a**) metastatic from non-metastatic patients and (**b**) between the combined genotypes of rs2067087 (*CC* vs. *GG* + *GC*). Blue stepwise curve: actual diagnostic performance of the panel; Green diagonal line: performance expected by random chance (no discrimination, AUC = 0.5).

Binary logistic regression analysis demonstrated that the panel of biomarkers significantly discriminated between metastatic and non-metastatic cases (*p* < 0.001, Omnibus test). The model showed a good fit (Hosmer–Lemeshow test, *p* = 0.84). The regression model yielded an overall classification accuracy of 72.3%, with a sensitivity of 97.1% and a specificity of 96.7% in identifying metastatic cases at a cut-off point of 0.176. The ROC curve analysis revealed an Area under the curve (AUC) of 0.723 (95% CI: 0.646–0.8), indicating good discriminative ability of the panel (Figure 4a).

Multinomial logistic regression analysis demonstrated that the panel significantly discriminated between the genotypes of rs2067087 (*p* = 0.042), indicating its potential utility in genotype differentiation for this variant. In contrast, no significant discrimination was observed among the genotypes of rs17501292 (*p* = 0.562), suggesting that the panel lacks predictive value for this particular SNP.

Moreover, binary logistic regression analysis demonstrated that the panel of biomarkers significantly discriminated between the combined genotypes of rs2067087 (*CC* vs. *GG* + *GC*) (*p* = 0.019, Omnibus test), confirming its potential utility in genotype differentiation for this SNP. This regression model yielded an overall classification accuracy of 62.3%, with a sensitivity of 98.1% and a specificity of 96.4% in identifying metastatic cases at a cut-off point of 0.314. The ROC curve analysis revealed an AUC of 0.6 (95% CI: 0.508–0.673), indicating acceptable discriminative ability of the panel (Figure 4b). The model failed to predict genotype group membership of rs17501292 and also failed to predict haplotype membership of both SNPs.

### 2.4. Association of HOTTIP Gene SNPs with the Risk of Metastasis

As represented in (Table 3), the *TT* genotype of the codominant genetic model of rs17501292 was associated with a significantly decreased risk of HCC (OR = 0.443, 95% CI = 0.167–1.178; *p* = 0.027), and the *TG* genotype of the codominant genetic model of rs17501292 was associated with a significantly decreased risk of HCC (OR = 0.997, 95% CI = 0.367–0.712; *p* = 0.027) compared with individuals carrying the *GG* genotype. The dominant genetic model of rs17501292 showed a significant 2.25-fold increase in the odds of metastasis (95% CI = 1.241–4.086, *p* = 0.007). Under the recessive genetic model of the same SNP, we failed to find a significant difference in the odds of metastasis. Additionally, a significantly decreased risk was observed for the *T* allele of rs17501292 (OR = 0.592, 95% CI = 0.373–0.942; *p* = 0.013) compared with the *G* allele.

In terms of rs2067087, the *GG* genotype of the codominant genetic model was associated with a significantly decreased risk of HCC (OR = 0.751, 95% CI = 0.265–2.122; *p* = 0.008), and the *GC* genotype of the codominant genetic model was associated with a significantly decreased risk of HCC (OR = 0.377, 95% CI = 0.2–0.711; *p* = 0.008) compared with individuals carrying the *CC* genotype. The dominant genetic model of rs2067087 showed a significant decrease in the odds of metastasis (OR = 0.426, 95% CI = 0.235–0.774, *p* = 0.005). Under the recessive genetic model of the same SNP, we failed to find a significant difference in the odds of metastasis, with a *p*-value of 0.708. Additionally, a significantly decreased borderline risk was observed in the *G* allele of rs2067087 (OR = 0.712, 95% CI = 0.447–1.135; *p* = 0.057) compared with the *C* allele. This finding was in agreement with the Hardy–Weinberg equilibrium (*P_HWE_* value > 0.05) in the whole population and in the individual study groups. The adjusted *p* values for the effects of the covariates of both age and gender, performed by GLM, are presented in (Table 3).

Additionally, we analyzed the relationship between each *HOTTIP* SNP and HCC metastasis via a Cox regression model by using groups as covariates. We found that rs17501292 was associated with a significantly lower risk of metastasis in the *TG* and *TT* subgroups (hazard ratio (HR) = 0.105, CI = 0.054–0.205, *p* < 0.0001) and (HR = 0.07, CI = 0.036–0.134, *p* < 0.0001), respectively, than in the *GG* subgroup. However, only the *GC* subgroup of rs2067087 had a significantly lower risk of metastasis (HR = 0.388, CI = 0.227–0.665, *p* < 0.0001) (Table 4).

### 2.5. Association of HOTTIP Gene SNPs with Disease Prognosis

We analyzed the associations of the studied SNPs with prognosis by classifying the patients into two groups on the basis of their APRI scores. Those with an APRI score lower than 0.5 had a favorable prognosis, whereas those with an APRI score of 0.5 or greater had an unfavorable prognosis. As shown in (Table 5), the *TT* genotype of the codominant genetic model of rs17501292 was associated with a significantly better prognosis (OR = 0.343, 95% C.I = 0.115–1.018; *p* = 0.041). Moreover, the *TG* genotype of the codominant genetic model of rs17501292 was associated with a significantly better prognosis (OR = 0.635, 95% CI = 0.203–0.991; *p* = 0.041) than the *GG* genotype. The dominant genetic model of rs17501292 was associated with a significantly better prognosis (OR = 0.491, 95% C.I = 0.274–0.878, *p* = 0.02). Under the recessive genetic model of the same SNP, we failed to find a significant difference in the odds of unfavorable prognosis (*p* = 0.151). Additionally, a significantly better prognosis was observed for the *T* allele of rs17501292 (OR = 0.559, 95% C.I = 0.347–0.901; *p* = 0.009) than for the *G* allele.

With respect to rs2067087, the *GG* genotype of the codominant genetic model was associated with a significantly better prognosis (OR = 0.947, 95% C.I = 0.319–2.81; *p* = 0.013). Additionally, compared with individuals with the *CC* genotype, those with the *GC* genotype in the codominant genetic model also had a significantly better prognosis (OR = 0.41, 95% C.I = 0.222–0.757; *p* = 0.013). The dominant genetic model of rs2067087 showed a significant decrease in the odds of an unfavorable prognosis (OR = 0.468, 95% C.I = 0.26–0.844, *p* = 0.014). Under the recessive genetic model of the same SNP, we failed to find a significant difference in the odds of unfavorable prognosis, with a *p*-value of 0.617. Moreover, no significant difference in the odds of prognosis was observed for the *G* allele of rs2067087 (*p* = 0.131) compared with the *C* allele. The adjusted *p*-values for the effect of the covariates of both age and gender, as determined by GLM, are presented in (Table 5).

Receiver operating characteristic (ROC) curves for evaluating the prognostic value of rs17501292 codominant model (*GG* vs. *TG* vs. *TT*) and the rs2067087 dominant model (*GG* + *GC* vs. *CC*) are shown in Supplementary Figure S1a and S1b, respectively. These curves revealed a significant (*p* value < 0.05) association between the rs17501292 codominant model (*GG* vs. *TG* vs. *TT*) or the rs2067087 dominant model (*GG* + *GC* vs. *CC*) and prognosis, with modest to poor sensitivity and specificity (sensitivity: 52.2% and 49.6% and specificity: 65.1% and 68.2%, respectively).

### 2.6. The Diagnostic Value of HOTTIP Gene SNPs in the Studied Groups

In terms of diagnosis, the ROC curve was used to discriminate between the HCC metastatic and non-metastatic groups. The ROC curve for the rs17501292 codominant model (*GG* vs. *TG* vs. *TT*) revealed a non-significant diagnostic value (AUC of 0.555, sensitivity of 44.9%, specificity of 70.5% and *p*-value of 0.177) (Supplementary Figure S1c).

The ROC curve also revealed a significantly moderate diagnostic value for rs17501292 dominant model (*GG* + *TG* vs. *TT*) with an AUC, diagnostic sensitivity, specificity and *p*-value of 0.6, 58%, 62% and 0.007, respectively (Supplementary Figure S1d). For rs2067087 codominant model (*GG* vs. *GC* vs. *CC*), the ROC curve also revealed a significantly moderate to weak diagnostic value, with an AUC of 0.618, a sensitivity of 66.7%, and a specificity of 55.8%. with a *p*-value of 0.002 (Supplementary Figure S1e).

Finally, the ROC curve for rs2067087 dominant model (*GG* + *GC* vs. *CC*) had a significant modest to poor diagnostic value, with an AUC of 0.604, a sensitivity of 56.5%, and a specificity of 64.3%, with a *p*-value of 0.005 (Supplementary Figure S1f).

### 2.7. Association of Single Nucleotide Polymorphisms in the HOTTIP Gene with Patient Survival

**Figure 5:**
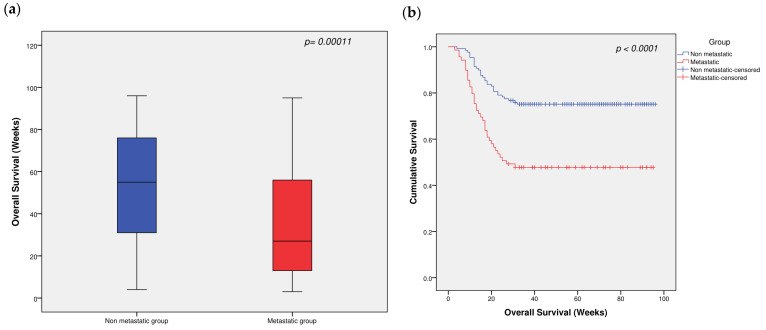

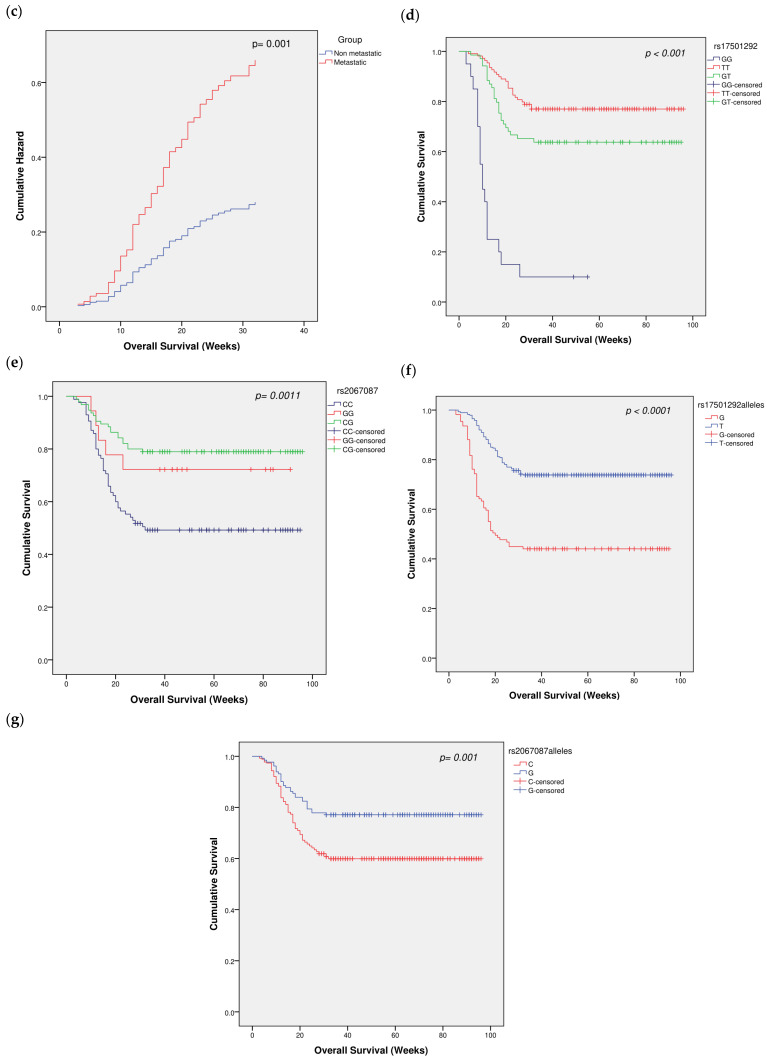
Box plots and Kaplan–Meier curves for patient groups with respect to OS: (**a**) Patient groups with respect to OS. Different groups are presented in box plots. The maximum and minimum values are represented by bars. (**b**) Kaplan–Meier estimates of OS according to group. Each censored case is represented by a (+) symbol. *p*-values were obtained from the log-rank (Mantel–Cox) test. (**c**) Cumulative survival function for both patient groups via the hazard function. Non-metastatic HCC, n = 129; metastatic HCC, n = 69. (**d**) rs17501292 genotypes. (**e**) rs2067087 genotypes. (**f**) rs17501292 alleles. (**g**) rs2067087 alleles. Each censored case is represented by a (+) symbol. *p*-values were obtained from the log-rank (Mantel–Cox) test.

In terms of patient survival, the metastatic group had significantly shorter OS than did the non-metastatic group (*p* = 0.00011, Figure 5a). Kaplan–Meier survival analysis revealed that the survival time of the metastatic group was significantly lower than that of the non-metastatic group (*p* < 0.0001, Figure 5b). Moreover, the cumulative survival function for both patient groups determined via the hazard function confirmed that the metastatic group had significantly lower survival, with a *p*-value of 0.001 (Figure 5c).

In addition, the *GG* genotype and *TG* genotype of rs17501292 had significantly lower survival time estimates than did the *TT* genotype (*p* < 0.001, Figure 5d), whereas the *CC* and *GG* genotypes of rs2067087 had significantly lower survival time estimates than did the *GC* genotype (*p* = 0.0011, Figure 5e). In terms of allele effects, the *G* allele had significantly lower survival time estimates than did the *T* allele (*p* < 0.0001, Figure 5f), whereas the *C* allele of rs2067087 had significantly lower survival time estimates than did the *G* allele (*p* = 0.001, Figure 5g).

Stratified Cox regression analyses were performed to evaluate the impact of *HOTTIP* polymorphisms on survival after adjustment for age, gender, treatment status (de novo vs. received therapy), and underlying liver disease etiology (HBV/HCV). For rs2067087, no significant association with overall survival was observed in the non-metastatic group (global Wald test, *p* = 0.295). Neither the *GG* genotype (HR = 1.83, 95% C.I: 0.85–3.93, *p* = 0.122) nor the *CC* genotype (HR = 1.54, 95% C.I: 0.44–5.43, *p* = 0.503) differed significantly from the GC reference genotype. Among the clinical covariates, age was the only independent predictor of survival in non-metastatic patients (HR = 0.95, 95% C.I: 0.90–0.99, *p* = 0.011).

In contrast, rs17501292 showed a strong independent association with overall survival in both metastatic and non-metastatic patients (global Wald test, *p* < 0.001 for both). In the metastatic group, compared with the TT genotype, carriers of the TG genotype exhibited a markedly increased risk of death (HR = 20.09, 95% C.I: 6.31–63.92, *p* < 0.001), whereas the *GG* genotype was associated with a significantly reduced hazard of death (HR = 0.41, 95% C.I: 0.18–0.94, *p* = 0.034). Age remained independently associated with survival in this group (HR = 0.95, 95% C.I: 0.90–0.99, *p* = 0.020).

Similarly, in non-metastatic patients, the TG genotype of rs17501292 was associated with significantly worse survival compared with the TT genotype (HR = 9.57, 95% C.I: 3.28–27.88, *p* < 0.001), while the *GG* genotype showed no significant effect (HR = 1.00, *p* = 0.997). No clinical covariates were independently associated with survival in this subgroup.

Interaction analysis using multivariate Cox regression revealed significant interactions between metastatic status and both *HOTTIP* polymorphisms. A significant interaction was observed between metastatic status and rs17501292 (HR = 0.40, 95% C.I: 0.22–0.75, *p* = 0.004), as well as between metastatic status and rs2067087 (HR = 0.36, 95% C.I: 0.22–0.59, *p* < 0.001), indicating that the associations between these variants and overall survival differed according to metastatic status. As expected, metastatic status itself was strongly associated with poor survival (HR = 19.42, 95% C.I: 7.07–53.36, *p* < 0.001). These effects remained significant after adjustment for age, gender, treatment status, and underlying liver disease etiology.

### 2.8. Results of Stratification Analysis

Age- and gender-based subgroup stratification analyses were used to determine the stratified effects of SNPs in lncRNAs on HCC risk. Logistic regression analysis revealed that there was no significant correlation between HCC metastasis risk and the age of genotype or allele frequency for rs17501292 (T/G), while gender showed a borderline significant effect on HCC metastasis risk, with a *p*-value of 0.045 (Table 6A). This applies only to rs2067087, where there is no significant correlation between HCC metastasis risk and the age of genotype and allele frequencies for rs2067087 (G/C), while gender showed a borderline significant effect on HCC metastasis risk, with a *p*-value of 0.047, as shown in (Table 6B).

### 2.9. Joint Effects and Results of Haplotype Analysis

Compared with those in the metastatic group, the combined impact of the investigated gene polymorphisms in patients with non-metastatic HCC was investigated (Table 7). The findings revealed that the GC haplotype (rs17501292 and rs2067087) was the only haplotype that protected against HCC metastasis (*GC* vs. *TG*, OR = 0.421; 95% CI = 0.233–0.76, *p* = 0.004). Furthermore, the combined heterozygosity of rs17501292 and rs2067087 (*TT* + *CC* vs. *TT* + *GG*, OR = 0.762; 95% CI = 0.198–2.938, *p* = 0.027) was a lower risk factor for HCC metastasis. This was also applied for combined heterozygosity (*TG* + *CC* vs. *TT* + *GG*, OR = 0.402; 95% CI = 0.106–1.522, *p* = 0.002), which was associated with a lower risk for HCC metastasis.

### 2.10. Results of Logistic Regression Analysis

It was found that rs17501292 (*GG* + *TG* vs. *TT*) and rs2067087 (*GG* + *GC* vs. *CC*) were identified as significant predictive factors for HCC metastasis risk in the univariate analysis, with adjustments for age and gender. rs17501292 (*TT* + *TG* vs. *GG*) and rs2067087 (*CC* + *GC* vs. *GG*) genotypes had non-significant predictive values (Table 8).

### 2.11. Molecular Docking Results

**Figure 6:**
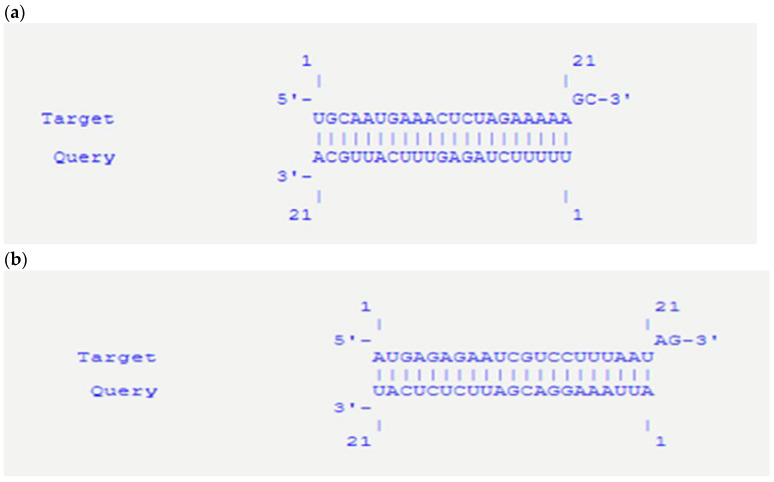
Interaction between certain positions on the target and guide siRNA: (**a**) Interaction between the 4169–4191 positions on the target and guide siRNAs. (**b**) Interaction between positions 4321–4343 on target and guide siRNAs.

The probabilities and docking scores for the siRNA population against HOTTIP are shown in (Table 9A). The targets with the best scores were chosen, where their 3D structure, energy, and hybridization energy were tabulated (Table 9B). Additionally, we verified that these siRNAs are the best siRNAs by ensuring that they can inhibit the direct interaction between HOTTIP and WDR5, as shown in (Table 10). Finally, these interactions can be seen in the bioinformatics results in (Figure 6a,b).

## 3. Discussion

This is the first study to investigate the minor allele frequency (MAF) and genotype distribution of the *HOTTIP* gene haplotype (rs17501292 (T/G) and rs2067087 (G/C)) and its association with metastasis in the Egyptian population of patients with HCC. The overall MAF of rs17501292 in our study was 0.28, which was slightly lower than that of the African population, which was 0.32 according to the 1000 Genomes Project [32]. With respect to the global population, a markedly lower MAF (0.19) was noted. This may be due to differences in ancestry and epigenetics among the Egyptian population. Moreover, the overall MAF of rs2067087 in our study was 0.33, which is slightly greater than that of the African population (0.27) according to the 1000 Genomes database, but very similar to that of the global population (0.32) [32]. Even while the MAFs reported in the present study were close to those reported for global or African populations, the genotype distributions of our study groups were distinct. Additionally, for the first time, in a population of Egyptian HCC patients, our study revealed a positive correlation between *HOTTIP* variants and metastasis.

Our study revealed that the CRP level was significantly greater in the metastatic group than in the non-metastatic group. This finding aligns with a previous study by Ma et al., who reported that the CRP serum level was significantly greater in HCC patients than in controls [33]. Another study revealed that certain cancer metastases can arise from inflammatory processes driven by adapted immune cells with elevated CRP levels [34].

The APRI score was significantly greater in the metastatic group than in the non-metastatic group. These findings support the findings of previous studies, indicating that the APRI score serves as a practical and independent prognostic indicator for late recurrence in HCC patients [35] and is a novel marker for assessing cirrhosis and survival in HCC patients, whose survival is worse in metastatic patients [36].

For tumor markers, the CEA level was also greater in the metastatic group than in the non-metastatic group, which is consistent with a recent study showing that patients with elevated CEA levels tend to have more aggressive disease progression, with an increased risk of metastasis to the LN and liver [37]. Finally, with respect to CA19.9 levels reported in the literature, elevated CA19.9 levels are associated with poor prognosis, worse outcomes, and higher mortality rates in HCC patients [38,39]. This can also be seen in our recent study, where CA19.9 was elevated in both groups but was significantly greater in the metastatic group. Moreover, this study is the first to evaluate a panel of both CEA and CA19.9 in discriminating metastatic from non-metastatic HCC patients.

Interestingly, our study is the first to explore the association between this haplotype and the risk of HCC metastasis. We found that the *TT* and *TG* genotypes of rs17501292 are significantly associated with a lower risk of HCC metastasis than the *GG* genotype. Moreover, the *T* allele is associated with a significantly lower risk of metastasis. On the other hand, the dominant genetic model revealed a significant 2.25-fold increased risk of metastasis, whereas under the recessive model, we failed to find a significant difference between the two groups. For rs2067087, individuals with the *GG* and *GC* genotypes have a significantly lower risk of HCC than individuals with the *CC* genotype. The dominant genetic model of rs2067087 revealed a significant decrease in the odds of metastasis, whereas under the recessive genetic model, we failed to find a significant difference in the odds of metastasis. Moreover, a significantly decreased borderline risk is observed in the *G* allele compared with the *C* allele of the same SNP. Furthermore, the combined effect of the investigated gene polymorphisms in patients with non-metastatic HCC, compared with the metastatic group, reveals that the *GC* haplotype (rs17501292 and rs2067087) is the only one offering protection against HCC metastasis. Additionally, the combined heterozygosity of rs17501292 and rs2067087 (*TT* + *CC* vs. *TT* + *GG*) is associated with a lower risk of HCC metastasis. Similarly, another combined heterozygosity pattern (*TG* + *CC* vs. *TT* + *GG*) also shows a reduced risk of HCC metastasis.

A subgroup stratification analysis based on age and gender was conducted to evaluate the stratified effects of SNPs in lncRNAs on HCC risk. Logistic regression analysis revealed no significant association between HCC metastasis risk and the genotype or allele frequency of rs17501292 (T/G) with respect to age. However, gender has a borderline significant effect on the risk of HCC metastasis. Similarly, for rs2067087 (G/C), no significant correlation was found between HCC metastasis risk and genotype or allele frequency in relation to age. However, gender again had a borderline significant effect on the risk of HCC metastasis. Univariate analysis adjusted for age and gender revealed that the rs17501292 dominant model (*GG* + *TG* vs. *TT*) and the rs2067087 dominant model (*GG* + *GC* vs. *CC*) were significant predictive factors for HCC metastasis risk. However, the predictive values for the rs17501292 recessive model (*TT* + *TG* vs. *GG*) and the rs2067087 recessive model (*CC* + *GC* vs. *GG*) genotypes were not significant. In agreement with our study, the only study concerning *HOTTIP* SNPs in HCC was in Chinese patients, where they revealed that, under allelic models, *HOTTIP* rs17501292, rs2067087, and rs17427960 SNPs increased the risk of HCC to 1.55-, 1.20-, and 1.18-fold that of control samples, respectively [30]. Another study concerning *HOTTIP* SNPs revealed that the expression of *HOTTIP* may be impacted by the rs2067087 and rs3807598 SNPs, which are linked to an increased risk of developing gastric cancer [40].

We also came to the same conclusion when we examined the relationship between the studied SNPs and patient prognosis. Compared with the *GG* genotype, the *TT* and *TG* genotypes for rs17501292 are significantly associated with better prognosis. Additionally, the dominant genetic model of the same SNP indicates a significantly better prognosis. However, no significant difference was found under the recessive genetic model. Compared with the *G* allele, the *T* allele is also associated with a significantly better prognosis. For rs2067087, the *GG* and *GC* genotypes are associated with a significantly better prognosis than the *CC* genotype. The dominant genetic model is associated with a reduced likelihood of an unfavorable prognosis. However, no significant association was found under the recessive genetic model, and the *G* allele did not significantly affect prognosis compared with the *C* allele (*p* = 0.131). ROC curves of the rs17501292 codominant model (*GG* vs. *TG* vs. *TT*) and the rs2067087 dominant model (*GG* + *GC* vs. *CC*) revealed moderately to weakly significant associations between these genotypes and patient prognosis. The observed AUC values, while statistically significant, indicate modest predictive ability and that the primary clinical relevance of the study lies in the prognostic associations identified through survival and interaction analyses rather than in the ROC performance of single genetic variants. This finding is consistent with a previous study that revealed that certain *HOTTIP* SNPs (rs3807598, rs17501292, rs2067087, and rs17427960) might be predictive biomarkers for the prognosis and risk of CRC [31]. This study provides insight into novel lncRNA-based genetic indicators to predict clinical outcomes and cancer risk.

For the diagnostic utility of SNPs, a receiver operating characteristic (ROC) curve was used to differentiate between the HCC metastatic and non-metastatic groups. The ROC analysis for the rs17501292 codominant model (*GG* vs. *TG* vs. *TT*) revealed a non-significant diagnostic value. In contrast, the ROC curve for the rs17501292 dominant model (*GG* + *TG* vs. *TT*) revealed significant diagnostic value. For the rs2067087 codominant model (*GG* vs. *GC* vs. *CC*), the ROC curve has significant diagnostic value, whereas the ROC curve for the rs2067087 dominant model (*GG* + *GC* vs. *CC*) has significant moderate diagnostic value. This may be attributed to the fact that SNP genotypes are categorical variables. This limited clinical significance is considered poor discriminatory ability; it cannot be a stand-alone factor to rely on while underscoring the need for integrative models combining genetic, clinical, and molecular variables. This finding is consistent with a study by Lv et al., which showed that *HOTTIP* SNPs might act as potential biomarkers for predicting CRC risk and, in turn, for CRC diagnosis [31].

In terms of survival outcomes, patients in the metastatic group had significantly shorter OS than did those in the non-metastatic group. Both Kaplan–Meier survival analysis and the cumulative survival function, analyzed through the hazard function, also revealed significantly lower survival time estimates for the metastatic group than for the non-metastatic group. Furthermore, patients with the *GG* and *TG* genotypes of rs17501292 have significantly shorter survival time estimates than those with the *TT* genotype. Similarly, the *CC* and *GG* genotypes of rs2067087 are associated with significantly lower survival time estimates than the *GC* genotype. Compared with the *T* allele, the *G* allele of rs17501292 is associated with significantly shorter survival time estimates, whereas the *C* allele of rs2067087 is linked to shorter survival time estimates. This finding aligns with the findings of a previous study demonstrating that rs17501292 enhances OS in CRC patients within the ulcerative/invasive tumor subtype subgroup [31].

The observed significant interactions between metastatic status and *HOTTIP* polymorphisms further support a context-dependent prognostic role of these variants in HCC. The differential survival effects of rs17501292 and rs2067087 according to metastatic status suggest that genetic variation within *HOTTIP* may modulate tumor behavior in a manner that becomes more pronounced or altered in the presence of metastatic disease. *HOTTIP* is known to influence transcriptional regulation and tumor progression pathways in HCC, and it is plausible that specific polymorphisms affect its regulatory function under distinct biological contexts [14]. While the present study does not address causality or treatment-specific effects, these interaction findings highlight the importance of considering metastatic status when evaluating the prognostic relevance of *HOTTIP* variants and may help guide future experimental and clinical investigations into their functional roles in cancer.

Regarding the potential functional mechanisms of *HOTTIP* rs17501292 and rs2067087, although rs17501292 and rs2067087 are located within the non-coding region of the lncRNA HOTTIP, accumulating evidence suggests that non-coding SNPs can exert functional effects through multiple regulatory mechanisms [41]. These include modulation of lncRNA expression levels, alteration of RNA secondary structure and stability, and disruption of interactions with protein partners or regulatory molecules such as transcription factors and miRNAs [41].

One plausible mechanism is that these variants influence *HOTTIP* transcriptional regulation by altering transcription factor binding motifs or chromatin accessibility at the *HOTTIP* locus [42]. Such effects could result in differential *HOTTIP* expression, which is biologically relevant given that HOTTIP overexpression has been consistently associated with enhanced tumor proliferation, invasion, and metastasis in HCC through epigenetic activation of downstream oncogenic pathways.

Additionally, non-coding SNPs within lncRNAs may affect RNA secondary structure, thereby influencing RNA stability and molecular interactions [43]. Structural alterations could modulate the affinity of HOTTIP for key protein partners, particularly WDR5, a core component of the MLL complex that mediates H3K4 trimethylation and transcriptional activation of metastasis-related genes [21]. In this context, protective genotypes identified in the present study may reduce the efficiency or stability of the HOTTIP-WDR5 interaction, leading to attenuated activation of oncogenic transcriptional programs.

Moreover, emerging evidence indicates that lncRNA polymorphisms can influence miRNA–lncRNA interactions, acting as competing endogenous RNAs and thereby indirectly regulating gene expression networks [44]. Although not directly assessed in the present study, rs17501292 and rs2067087 may alter putative miRNA binding sites within *HOTTIP*, contributing to differential post-transcriptional regulation. This possibility warrants further investigation using dedicated functional assays.

The pathway enrichment analysis performed in this study further supports this hypothesis, as genes associated with *HOTTIP* were significantly enriched in pathways related to cell proliferation, apoptosis, migration, and metastasis. These pathways are highly relevant to HCC progression and provide a functional framework linking *HOTTIP* genetic variation to the observed clinical outcomes. The concordance between enrichment results and clinical associations strengthens the biological plausibility of the identified SNPs as modulators of HCC metastasis and prognosis.

The molecular docking analysis employed a comprehensive computational approach to design siRNAs targeting the lncRNA HOTTIP to inhibit its interaction with the WDR5 protein. The interaction between HOTTIP and WDR5 has been implicated in various cellular processes, and disrupting this interaction may have therapeutic potential in certain diseases [21]. By combining sequence-based interaction prediction, siRNA design and evaluation, interaction validation, and molecular docking, we identified two promising siRNA candidates that can potentially inhibit the HOTTIP–WDR5 interaction. The interaction prediction via catRAPID omics provides a foundation for identifying potential siRNA target sequences on HOTTIP that can disrupt its binding to WDR5. This step is crucial in narrowing the regions of interest on the lncRNA for further investigation. siDirect was then used to generate possible siRNA sequences targeting these regions, and each siRNA was evaluated via catRAPID omics for its potential to disrupt the HOTTIP–WDR5 interaction. This two-step process allows the identification of the most promising siRNA candidates.

Interaction validation via IntaRNA and molecular docking techniques provided further support for the selected siRNA candidates. IntaRNAs were used to calculate the interaction free energy (ΔG) between the siRNA targets and their guide sequences, confirming the stability of these interactions. Molecular docking via HNAdock and HDock allowed visualization of the 3D structures of the siRNA-HOTTIP target sequences and their guide sequences, as well as docking between the siRNA-HOTTIP complex and WDR5. These analyses provided additional confidence in the ability of the selected siRNAs to inhibit the HOTTIP–WDR5 interaction. The two best siRNA candidates, which target positions 4321–4343 and 4169–4191 on HOTTIP, demonstrated favorable specificity and stability properties. Both siRNAs show a minimum of two mismatches against off-target effects, reducing the likelihood of unintended effects on other genes. Additionally, their seed-duplex stability (Tm) values of 11.0 °C and 7.1 °C suggest that these siRNAs will form stable complexes with their targets, increasing their potential efficacy.

Importantly, the molecular docking and siRNA-based computational analyses performed in this study provide mechanistic insight by demonstrating that disruption of the HOTTIP–WDR5 interaction is structurally feasible. These findings support a model in which genetic variation within *HOTTIP* may influence tumor behavior by modulating its interaction with epigenetic regulators and downstream signaling pathways implicated in HCC metastasis. Furthermore, this study highlights the potential of a rational, computational approach to design siRNAs that specifically target lncRNA–protein interactions. By identifying two promising siRNA candidates capable of inhibiting the HOTTIP–WDR5 interaction, this work lays the foundation for future experimental studies to explore their therapeutic potential. The comprehensive methodology employed here can also be applied to other lncRNA–protein interactions, facilitating the discovery of novel therapeutic targets and strategies.

Limitations. The current study has the following limitations: first, studies investigating the interplay between *HOTTIP* and adipokine-related genetic variants or expression levels and inflammatory markers within the tumor immune microenvironment are lacking [45]. Additionally, exploring the modulatory effects of vitamins such as vitamin E and vitamin D [46] on the tumor microenvironment, as well as the involvement of insulin resistance [47], insulin-like growth factors [48], and other metabolism-related hormones or genes across various noncommunicable diseases [1,48,49,50,51,52,53] could provide deeper insights. Second, the study was conducted at a single center and focused exclusively on Egyptian patients, which may affect generalizability to other populations. Moreover, the absence of healthy control subjects precluded baseline comparisons for some biomarkers. Third, the need for Functional Validation: Although associations between *HOTTIP* SNPs and HCC metastasis/survival we observed, the study requires experimental validation (e.g., CRISPR/Cas9 knockdown, or siRNA transfection in cell lines) to confirm the functional impact of the identified polymorphisms. Additionally, the lack of detailed information on treatment modalities represents a limitation of this study and precluded assessment of treatment-specific survival effects. Finally, in Silico siRNA Design Without Experimental Testing: While molecular docking and computational siRNA design against HOTTIP–WDR5 interactions are innovative, these findings remain theoretical. No in vitro or in vivo assays were performed to test the efficacy or specificity of the designed siRNAs, which limits mechanistic interpretation. Despite these limitations, our study provides a valuable framework that can guide future experimental and clinical investigations into the role of *HOTTIP* in cancer in multi-center studies on higher sample sizes.

## 4. Materials and Methods

### 4.1. Bioinformatic Analysis

#### 4.1.1. DGE of Different Genes from Online Datasets in Liver Cancer

To retrieve relevant gene expression data, we accessed the UCSC Xena browser (https://xenabrowser.net, accessed on 15 November 2024), selected the dataset (TCGA RNA-Seq data), and then identified the cancer type “TCGA liver cancer (LIHC)”. Samples were selected based on clinical features (primary tumor vs. solid normal tissue) to compare the expression of different genes in available online datasets via the Xena DGE Analysis Pipeline (https://github.com/ucscXena, accessed on 17 November 2024) (adapted from the Ma’ayan laboratory’s Appyter bulk RNA-seq analysis https://appyters.maayanlab.cloud/#/Bulk_RNA_seq, accessed on 17 November 2024) to perform DGE analysis and further downstream analyses.

#### 4.1.2. Principal Component Analysis

To detect overarching patterns in high-dimensional data to assess the similarity between biological samples in gene expression studies, the Xena differential gene expression analysis pipeline was used.

#### 4.1.3. Pathway Enrichment Analysis

To determine whether a set of genes shows statistically significant, non-random associations with predefined biological pathways/functional categories, the Kyoto Encyclopedia of Genes and Genomes (KEGG) database in LIHC was used. This was followed by Enrichr use to determine which biological pathways were significantly enriched among the upregulated and downregulated genes in LIHC. For GO, we selected GO Categories for analysis and chose the Molecular Function category via the Xena Differential Gene Expression Analysis Pipeline.

#### 4.1.4. PPI and Gene–Protein Interaction

The HOTTIP interaction network obtained from BioGRID^4.4^ (https://thebiogrid.org/, accessed on 21 April 2025) was used to identify its downstream targets.

#### 4.1.5. Selected Polymorphic Sites in the lncRNA HOTTIP

Polymorphisms were identified via data from the 1000 Genomes Project (http://www.internationalgenome.org/home, accessed on 10 January 2022), as outlined in previous studies [54,55,56]. Tagging SNPs (tagSNPs) were selected separately based on the following criteria: (1) Haploview software (V 4.2) with the Tagger function was utilized; (2) the Yoruba population in Ibadan, Nigeria (YRI) from the HapMap project was chosen; (3) SNPs with a pairwise tagging r^2^ of ≥ 0.8 were included; and (4) those with a minor allele frequency of ≥5% were prioritized. The selection region was expanded 10 kb upstream and downstream of the *HOTTIP* lncRNA gene. To predict the functional potential of the SNPs, functional annotation tools including the SNPinfo Web Server (FuncPred module) and Ensembl Variant Effect Predictor (VEP) were employed (https://snpinfo.niehs.nih.gov/, https://www.ensembl.org/vep, accessed on 11 January 2022) [57,58].

### 4.2. Sample Size and Power Study

The sample size calculation was based on a prior study by Wang et al. (2018), which employed an unmatched case-control design (1:1.5 ratio) with an OR of 2.46 [30]. The population risk of developing HCC was estimated at 0.12 (kp = 0.12) based on literature data, and the allele frequency was set at 0.48. Sample size estimation was conducted via QUANTO 1.2.4 software [59], with a two-sided confidence level of 95%. To achieve a statistical power of 0.95 and a type I error probability of 0.05, the required sample size was determined to be 169 participants. To account for potential errors or sample loss, the final sample size increased by almost 15% to 198 participants.

### 4.3. Clinical and Pathological Characteristics of HCC Patients

A total of 198 HCC patients were recruited from the Oncology Treatment and Nuclear Medicine Department, Faculty of Medicine at Ain Shams University Hospitals from 20th of May 2022 until 22nd of August 2023. These patients were divided into two groups: Group 1 (n = 129) included non-metastatic HCC patients, and Group 2 (n = 69) included metastatic HCC patients. All participants had primary HCC and were receiving treatment, including neoadjuvant therapy or radiotherapy. The eligibility criterion included adult patients of either gender. The diagnosis of HCC was established based on internationally accepted diagnostic guidelines, primarily based on characteristic radiological features observed on contrast-enhanced computed tomography (CT), including arterial phase hyperenhancement with portal venous and/or delayed phase washout. Serum alpha-fetoprotein (AFP) levels were used as a supportive biomarker but were not considered a sole diagnostic criterion. In cases with atypical or inconclusive imaging findings, histopathological confirmation was obtained from pathology reports. The participants were matched by socioeconomic status, age range, and residence in this case-controlled study.

Patients were classified as having metastatic HCC if radiological evidence, mainly Positron Emission Tomography (PET) and CT (PET-CT) of distant organs, showed involvement or extrahepatic metastatic lesions were detected. Patients were classified as non-metastatic HCC when no radiological or clinical evidence of extrahepatic metastasis was present at the time of enrollment. The exclusion criteria included a history of liver transplantation, concurrent cancers, renal insufficiency, thyroid dysfunction, incomplete medical records, or missing histopathology diagnoses.

Clinical and pathological data were collected from medical records and pathology reports and compiled into an Excel sheet. These data included histological grading of HCC according to the Edmondson–Steiner (ES) criteria [60]. Child–Pugh scoring for cirrhosis, ultrasonography (U.S.) findings, and various laboratory results: total and direct bilirubin, alkaline phosphatase (ALP), prothrombin time (PT), international normalized ratio (INR), complete blood count (CBC), random blood glucose levels, and albumin. Tumor size and clinicopathological biomarkers, including AFP and gamma-glutamyl transferase (GGT), were also documented for statistical analysis and correlations.

For the metastatic group, data regarding metastatic sites were collected. For both groups, OS data, defined as the duration of survival from the initiation of treatment, were included as a standard metric for clinical benefit [61].

### 4.4. Blood Samples

Following standard biosecurity and international safety protocols, eight milliliters of peripheral venous blood were drawn from HCC patients (either metastatic or not) and divided into 2 vacutainers (plain and ethylenediaminetetraacetic acid (EDTA)). For plain vacutainers, centrifugation was carried out for 20 min at 4000 rpm at 4 °C after allowing for clotting for 15 min at room temperature (RT). Once needed, the sera were stored at −80 °C after being aliquoted into four sterile, nuclease-free tubes. EDTA vacutainers were used for genomic DNA extraction from all the subjects (kept as such and stored at −20 °C until the DNA extraction step).

### 4.5. Routine Blood Measurements

The demographic and clinical parameters, such as age, gender, total and direct bilirubin, aspartate transaminase (AST), alanine transaminase (ALT), albumin, serum creatinine, INR, AFP, random blood glucose (RBG), and CBC, were gathered from patients’ files. Other missing routine measurements were analyzed using separated sera. These include C-reactive protein (CRP), traditional protein tumor markers, carcinoembryonic antigen (CEA), cancer antigen 19.9 (CA19.9), Triglycerides (TG), and total cholesterol (TC). All the experiments were performed via the corresponding kit (from Quanita, Verna, India/Monocent, Canoga Park, USA/Pishtaz, Tehran, Iran/Biodiagnostic, Giza, Egypt/Biodiagnostic, Giza, Egypt, respectively) and according to the manufacturer’s instructions. Finally, the APRI score was calculated via the following formula: [APRI = (AST/upper limit of normal) × 100/platelet count].

### 4.6. DNA Extraction

Genomic DNA was isolated from whole blood samples collected in EDTA tubes from all participants via the QIAamp DNA Mini Kit (Qiagen, Valencia, CA, USA) following the manufacturer’s protocol. The DNA yield was quantified via a NanoDrop 2000 spectrophotometer (Thermo Fisher Scientific, Waltham, MA, USA). The extracted DNA was aliquoted into three clean Eppendorf tubes and stored at −20 °C until further biochemical analysis at the Faculty of Pharmacy, Ain Shams University.

### 4.7. SNPs Genotyping

Two SNPs, part of a haplotype—rs17501292 (NC_000007.14:27201853:T:C, NC_000007.14:27201853:T:G; T > C, G) and rs2067087 (NC_000007.14:27202040:G:C, NC_000007.14:27202040:G:T; G > C, T) were genotyped (Thermo Fisher Scientific Inc., Massachusetts, USA). This was performed via real-time polymerase chain reaction (RT-PCR) with the TaqMan allelic discrimination assay on a 7900 system, which employs predesigned primers and probes provided by Applied Biosystems, Inc., via a StepOne Plus RT-PCR device (Applied Biosystems, Foster City, CA, USA).

### 4.8. Molecular Docking Study

Potential drugs that could be repurposed to inhibit our target oncogenic lncRNA HOTTIP were explored.

#### 4.8.1. In Silico Molecular Modeling Study: Sequence Retrieval

The sequence of the lncRNA HOTTIP was retrieved from the National Center for Biotechnology Information (NCBI) database (https://www.ncbi.nlm.nih.gov, accessed on 15 September 2024) via accession number NR_037843.3, and its major downstream target was retrieved from the UniProt (https://www.uniprot.org, accessed on 15 September 2024) database.

#### 4.8.2. Interaction Prediction

The probability of interaction between the lncRNA HOTTIP and its downstream target protein was assessed *in silico* using the catRAPID omics v2.0 web server for RNA-protein interaction prediction (sequence based interaction propensity analysis, available at (https://service.tartaglialab.com/page/catrapid_omics2_group, accessed on 16 September 2024) [62] was employed to assess the probability of interaction between the lncRNA HOTTIP and its downstream target protein.

#### 4.8.3. siRNA Design and Evaluation

The siDirect (siRNA Design and Rational Evaluation) [63] tool was utilized to generate all possible siRNA sequences targeting the lncRNA HOTTIP, aiming to inhibit its interaction(s). Each resulting siRNA target sequence was evaluated for its potential to disrupt the HOTTIP–protein/gene interaction via catRAPID omics v2.0 web server.

#### 4.8.4. Interaction Validation

IntaRNA (Interaction Prediction for RNA–RNA Interactions) (https://rna.informatik.uni-freiburg.de/IntaRNA/, accessed on 20 September 2024) [64] was employed to validate the interaction between the selected siRNA targets and their corresponding guide sequences by calculating the interaction free energy (ΔG).

#### 4.8.5. Molecular Docking

The 3D structures of the siRNA-HOTTIP target sequences and their respective guide sequences were predicted via RNA Composer (https://rnacomposer.cs.put.poznan.pl/ accessed on 20 September 2024) [65], and then, molecular docking between the siRNA-HOTTIP target sequences and their corresponding guide sequences was performed via the HNADock webserver (http://huanglab.phys.hust.edu.cn/hnadock/ accessed on 20 September 2024) [66]. The docking results were further validated via the HDock webserver [67]. The 3D structures of the full-length lncRNA HOTTIP and its downstream protein were predicted via AlphaFold3 (https://alphafoldserver.com/ accessed on 21 September 2024) [68] to validate the interaction positions. Molecular docking between the siRNA-HOTTIP complex and the target protein was carried out via HDock to validate the overall experimental design.

### 4.9. Statistical Analysis

Statistical analyses were performed via SPSS version 23, a software package for social sciences (IBM, Armonk, NY, USA). The data were tested for normality via the Shapiro–Wilk test.

Data with a normal distribution is presented as the mean ± S.E.M or, where appropriate, as numbers and percentages (n,%). Non-parametric data are summarized as medians with interquartile ranges (25th–75th percentiles). Categorical data comparisons were conducted via Fisher’s exact test or the χ^2^ test, as applicable. Continuous variables were compared via Student’s *t*-test or one-way analysis of variance (ANOVA), followed by Tukey’s post hoc test when needed. Consistent with the known heterogeneity of HCC, AFP values were highly skewed within the same group of patients. To address this and improve statistical robustness, AFP values were log10-transformed before analysis, and the transformed values were used in subsequent analyses.

To assess the ability of selected common tumor biomarkers (CEA and CA19.9) as a panel to discriminate between metastatic and non-metastatic cases, we performed a binary logistic regression analysis. The overall significance of the regression model was evaluated using the Omnibus Test of Model Coefficients, while model fit was assessed via the Hosmer–Lemeshow goodness-of-fit test. The predictive performance of the panel was further evaluated by generating ROC curves using the predicted probabilities obtained from the logistic model. AUC was calculated to assess the discriminative power of the biomarker panel. Classification accuracy, sensitivity, and specificity were derived from the classification table and ROC analysis. Statistical significance was set at *p* < 0.05. We also assessed the ability of this panel to discriminate between different variants of our target SNPs via multinomial logistic regression.

Treatment status (de novo vs. previously treated HCC) was included as a covariate/confounding factor in univariate and multivariate Cox proportional hazards regression analyses, together with age, gender, and HBV/HCV status, to exclude their effects on OS and thus survival analysis. Additionally, to assess whether the association between *HOTTIP* polymorphisms and overall survival differed according to metastatic status, interaction analyses were performed using multivariate Cox proportional hazards models. Interaction terms were generated by multiplying metastatic status (metastatic vs. non-metastatic) with each SNP genotype variable (rs17501292 and rs2067087). These interaction terms were included in Cox regression models together with the corresponding main effects and adjusted for age, gender, treatment status (de novo vs. previously treated), and underlying liver disease etiology (HBV vs. HCV). HRs with 95% CIs were estimated, and the statistical significance of interaction effects was assessed using Wald tests.

The relationships between genotypes and the risk of HCC metastasis were evaluated through logistic regression analysis, and ORs with 95% CIs were calculated. Prognostic factors were analyzed via both univariate and multivariate logistic models. Significant predictors identified in the univariate analysis (*p* < 0.05) were further evaluated through stepwise forward multivariate analysis. ROC curve analysis was performed via MedCalc software (version 20.0).

## 5. Conclusions

This study is the first to explore the MAF, genotype distribution, prognosis, and survival of patients with *HOTTIP* gene polymorphisms (rs17501292 and rs2067087) and their associations with metastasis in HCC patients. The findings reveal distinctive MAFs and genotype distributions compared with those of African and global populations, likely due to unique genetic ancestry. The results identified specific genotypes and haplotypes of these SNPs as significant predictive factors for the risk of HCC metastasis, with the TT and TG genotypes of rs17501292 and the *GG* and *GC* genotypes of rs2067087 showing a reduced risk of metastasis. Additionally, combined haplotype analysis highlighted the protective effect of specific genetic patterns. The study also underscores the clinical relevance of inflammatory markers such as CRP, APRI scores, and tumor markers (CEA, CA19.9) in distinguishing metastatic from non-metastatic groups. These markers align with previous findings that link inflammation and advanced cancer stages. Survival analysis further revealed significant associations between SNP genotypes and prognosis, with genotypes such as TT (rs17501292) and *GC* (rs2067087) correlating with better survival outcomes.

For diagnostic and prognostic purposes, ROC curve analysis demonstrated that specific genetic models of rs17501292 and rs2067087 have significant diagnostic and prognostic value(s). These findings are consistent with previous studies on *HOTTIP* SNPs in other cancers, suggesting their utility as potential biomarkers for metastasis and survival prediction. Moreover, the study incorporated a molecular docking approach to design siRNAs targeting HOTTIP–WDR5 interactions. This innovative computational strategy identified two promising siRNA candidates, highlighting the therapeutic potential of disrupting lncRNA–protein interactions in HCC. Moreover, whether these could be targeted for tumor delivery via innovative treatment strategies [69,70] constitutes an opportunity for examination.

This research provides novel insights into the epigenetic and molecular underpinnings of HCC metastasis in the Egyptian population. These findings highlight the prognostic and therapeutic significance of *HOTTIP* SNPs and establish a foundation for future experimental studies to validate these findings and explore more targeted therapies. The integration of epigenetic analysis and computational design provides a comprehensive approach for understanding and combating HCC metastasis.

**Figure 7:**
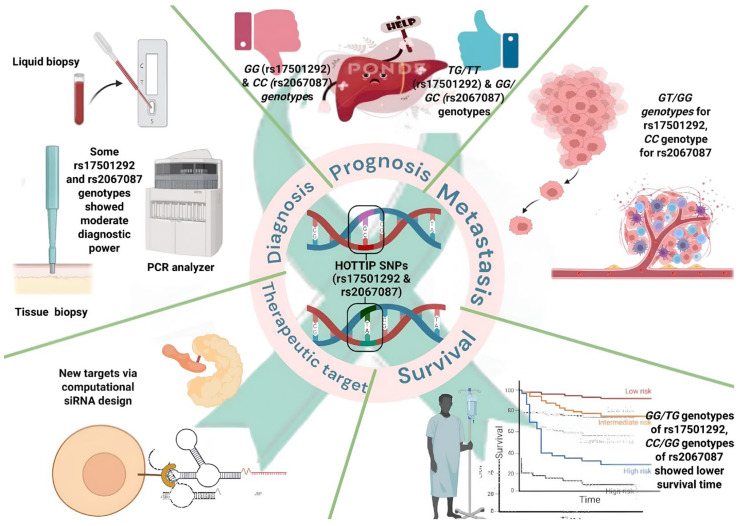
Graphical representation of the workflow abstract [Created in BioRender. Galal, M. (2025) https://BioRender.com/p73xo8d].

The role of *HOTTIP* haplotype in HCC can be summarized in (Figure 7).

Online Databases used:

- UCSC Xena Browser: (https://xenabrowser.net, accessed on 15 November 2024).
- Xena Differential Gene Expression Analysis Pipeline: (https://github.com/ucscXena, accessed on 17 November 2024) (adapted from the Ma’ayan lab’s Appyter bulk RNA-seq analysis https://appyters.maayanlab.cloud/#/Bulk_RNA_seq, accessed on 17 November 2024).
- BioGRID^4.4^: (https://thebiogrid.org/, accessed on 21 April 2025).
- 1000 Genomes Project: (http://www.internationalgenome.org/home, accessed on 10 January 2022).
- SNPinfo Web Server (FuncPred module) and Ensembl Variant Effect Predictor (VEP): (https://snpinfo.niehs.nih.gov/, https://www.ensembl.org/vep, accessed on 11 January 2022).
- NCBI database: (https://www.ncbi.nlm.nih.gov, accessed on 15 September 2024).
- UniProt: (https://www.uniprot.org, accessed on 15 September 2024).
- catRAPID omics v2.0 web server: (https://service.tartaglialab.com/page/catrapid_omics2_group, accessed on 16 September 2024).
- IntaRNA: (https://rna.informatik.uni-freiburg.de/IntaRNA/, accessed on 20 September 2024).
- RNA Composer: (https://rnacomposer.cs.put.poznan.pl/, accessed on 20 September 2024).
- HNAdock webserver: (http://huanglab.phys.hust.edu.cn/hnadock/, accessed on 20 September 2024).
- AlphaFold3: (https://alphafoldserver.com/, accessed on 21 September 2024).

## Figures and Tables

**Table 1:** Clinical and laboratory characteristics of the studied groups.

Factor	Non-Metastatic	Metastatic	*p*-Value	*p*-Value ^¶^
N (M/F)	129 (84/45)	69 (54/15)	-	-
Age (Year)	63.28 ± 0.703	62.29 ± 0.915	NS ^1^	-
Treatment status (D/T)	129 (73/56)	69 (30/39)	NS ^1^	0.015
CPS (A5 + A6/B7 + B8 + B9 + C10)	129 (92/37)	69 (51/18)	NS ^1^	NS
HTN (Y/N)	129 (53/75)	69 (29/40)	NS ^1^	NS
DM (Y/N)	129 (44/85)	69 (25/44)	NS ^1^	NS
Smoking (Y/N)	129 (15/114)	69 (9/60)	NS ^1^	NS
Vascularization (Y/N)	129 (46/83)	69 (30/39)	NS ^1^	NS
Cause (HBV/HCV)	129 (7/122)	69 (9/60)	NS ^1^	NS
Ascites (Y/N)	129 (48/81)	69 (31/38)	NS ^1^	NS
T.Bil (mg/dL) ^a^	1.14 ± 0.05	1.09 ± 0.09	NS ^1^	NS
D.Bil (mg/dL) ^a^	0.45 ± 0.04	0.45 ± 0.06	NS ^1^	NS
ALT (IU/L) ^a^	43.77 ± 3.14	44 ± 3.58	NS ^1^	NS
AST (IU/L) ^a^	62.95 ± 3.24	65.33 ± 7.49	NS ^1^	NS
CRP (mg/L) ^a^	11.88 ± 1.58	17.9 ± 3.58	0.047 ^1^	NS
Albumin (g/dL) ^a^	3.55 ± 0.052	3.59 ± 0.063	NS ^1^	NS
Creatinine (mg/dL) ^a^	0.95 ± 0.024	1.1 ± 0.089	0.04 ^1^	NS
INR ^a^	1.19 ± 0.015	1.15 ± 0.019	NS ^1^	NS
PLT (×10^3^/uL) ^a^	162.09 ± 8.38	199.81 ± 12.54	0.014 ^1^	0.024
APRI Score ^a^	1.09 ± 0.14	1.299 ± 0.088	<0.001 ^1^	0.008
Log10 (AFP) ^a^	2.33 ± 0.13	2.28 ± 0.16	NS ^1^	NS
CEA (ng/mL) ^a^	5.5 ± 0.39	9.18 ± 1.3	0.001 ^1^	0.003
CA19.9 (U/mL) ^a^	23.29 ± 2.39	58.82 ± 10.53	<0.001 ^1^	0.001
RBG (mg/dL) ^a^	148.42 ± 8.28	157.62 ± 13.39	NS ^1^	NS
TC (mg/dL) ^a^	168.19 ± 5.72	192.28 ± 9.85	0.037 ^1^	NS
TG (mg/dL) ^a^	134.21 ± 5.93	141.12 ± 9.29	NS ^1^	NS
WBCs (×10^3^/uL) ^a^	5.98 ± 0.27	6.23 ± 0.29	NS ^1^	NS
Neut (×10^3^/uL) ^a^	3.88 ± 0.21	3.81 ± 0.21	NS ^1^	NS
Lym (×10^3^/uL) ^a^	1.47 ± 0.07	1.69 ± 0.11	NS ^1^	NS
MO (×10^3^/uL) ^a^	0.5 ± 0.027	0.56 ± 0.039	NS ^1^	NS
EO (×10^3^/uL) ^a^	0.11 ± 0.008	0.16 ± 0.017	0.013 ^1^	0.027
BA (×10^3^/uL) ^a^	0.021 ± 0.003	0.022 ± 0.004	NS ^1^	NS
RBCs (×10^6^/uL) ^b^	4.13 (3.63–4.72)	4.34 (3.78–4.96)	0.048 ^2^	- ^3^
HGB (g/dL) ^b^	11.9 (10.5–13.4)	11.8 (10.6–13.5)	NS ^2^	- ^3^
HCT% ^b^	35.7 (32.55–39.95)	35 (31.5–40.6)	NS ^2^	- ^3^
MCV (fl) ^b^	86.8 (81–92.44)	80.8 (77.15–87.85)	<0.001 ^2^	- ^3^
MCH (pg) ^b^	28.7 (26.15–30.6)	27.6 (26.25–29.14)	0.03 ^2^	- ^3^
MCHC (g/dL) ^b^	33.6 (32.3–34.75)	32.9 (32.1–34.1)	0.029 ^2^	- ^3^
RDW% ^a^	15.83 ± 0.338	15.64 ± 0.217	NS ^1^	NS

^a^ Results are expressed as the mean ± Standard error of mean (S.E.M). ^b^ Results are expressed as Median (25th quartile-75th quartile). [Status (D/T), (De Novo/Therapy); CPS, Child-Pugh Score; HTN: Hypertension; DM, diabetes mellitus; T.Bil, Total Bilirubin; D.Bil, Direct Bilirubin; PLT, Platelets; RBG, Random Blood Glucose; TC, Total Cholesterol; TG, Triglycerides; WBCs: White Blood Cells; Neut, Neutrophils; Lym, Lymphocytes; MO, Monocytes; EO, Eosinophils; BA, Basophils; RBCs, Red Blood Cells; HGB, Hemoglobin; HCT%, Hematocrit%; MCV, Mean Corpuscular Volume; MCH, Mean Corpuscular Hemoglobin; MCHC, Mean Corpuscular Hemoglobin Concentration; RDW, Red cell Distribution Width%; NS, non-significant]. AFP was originally measured in ng/mL, while AFP values were log10-transformed before analysis. ^1^ Independent-sample *T*-test, two-tailed, *p*-value > 0.05 non-significant. ^2^ Mann–Whitney U test, two-tailed, *p*-value > 0.05 non-significant. ^3^ Log- transformed for performing GLM but found to be nonlinear. ^¶^ *p*-value after adjustment for age and gender by GLM.

**Table 2:** Clinical characteristics of patients evaluated via ultrasonography.

Liver U.S.		Non-Metastatic (n = 129)	Metastatic (n = 69)	*p*-Value *	*p*-Value ^¶^
		n (%)	n (%)		
Tumor site	RT Lobe	67 (51.9%)	42 (60.9%)	NS	NS ^¶^
	Lt Lobe	17 (13.2%)	6 (8.7%)		
	Caudate Lobe	43 (33.3%)	1 (1.4%)		
	Bilobar	2 (1.6%)	20 (29%)		
PV	Patent	53 (41.1%)	34 (49.3%)	NS	NS ^¶^
	Thrombosed	76 (58.9%)	35 (50.7%)		
Metastasis site	None	129 (100%)	-	<0.001	<0.001 ^¶^
	Kidney	-	18 (26.1%)		
	Far LN	-	6 (8.7%)		
	Lung	-	19 (27.6%)		
	Bone	-	14 (20.3%)		
	Colon	-	7 (10.1%)		
	Prostate	-	5 (7.2%)		
Abdominal LN	Absent	79 (61.2%)	31 (44.9%)	0.029	NS ^¶^
	Present	50 (38.8%)	38 (55.1%)		

[U.S., Ultrasonography; RT lobe, Right lobe; Lt lobe, Left lobe; PV, Portal Vein; LN, Lymph Node; NS, non-significant.] * Independent-sample *T*-test, two-tailed, *p*-value > 0.05, non-significant. ^¶^ *p*-value after adjustment for age and gender by GLM.

**Table 3:** Association of *HOTTIP* gene SNPs with the risk of metastasis.

SNP	Genetic Model	Genotypes or Alleles	Non-Metastatic n (%)	Metastatic n (%)	*p*-Value	OR (95%CI)	*p*-Value ^¶^	*P*_HWE_ Value
rs17501292	Codominant	TT	80 (62.02%)	29 (42.03%)	0.027	0.443 (0.167–1.178)	0.015 ^¶^	NS
		TG	38 (29.45%)	31 (44.93%)		0.997 (0.367–0.712)		
		GG	11 (8.53%)	9 (13.04%)		1 (Ref.)		
	Dominant	GG + TG vs. TT	49 (37.98%) vs. 80 (62.02%)	40 (57.97%) vs. 29 (42.03%)	0.007	2.252 (1.241–4.086) vs. 1 (Ref.)	0.006 ^¶^	
	Recessive	TT + TG vs. GG	118 (91.47%) vs. 11 (8.53%)	60 (86.96%) vs. 9 (13.04%)	NS	0.621 (0.244–1.582) vs. 1 (Ref.)	NS ^¶^	
	-	T allele vs. G allele	198 (76.74%) vs. 60 (23.26%)	89 (64.49%) vs. 49 (35.51%)	0.013	0.592 (0.373–0.942) vs. 1 (Ref.)	0.03 ^¶^	
rs2067087	Codominant	GG	11 (8.53%)	7 (10.14%)	0.008	0.751 (0.265–2.122)	0.004 ^¶^	NS
		GC	72 (55.81%)	23 (33.34%)		0.377 (0.2–0.711)		
		CC	46 (35.66%)	39 (56.52%)		1 (Ref.)		
	Recessive	CC + GC vs. GG	118 (91.47%) vs. 11 (8.53%)	62 (89.86%) vs. 7 (10.14%)	NS	1.211 (0.447–3.28) vs. 1 (Ref.)	NS ^¶^	
	Dominant	GG + GC vs. CC	83 (64.34%) vs. 46 (35.66%)	30 (43.48%) vs. 39 (56.52%)	0.005	0.426 (0.235–0.774) vs. 1 (Ref.)	0.005 ^¶^	
	-	G allele vs. C allele	94 (36.43%) vs. 164 (63.57%)	37 (26.81%) vs. 101 (73.19%)	NS	0.712 (0.447–1.135) vs. 1 (Ref.)	NS ^¶^	

A χ2 test was performed for various genotypes, the T allele, the G allele, and C allele. The odds ratio (OR) was calculated via binary logistic regression with metastasis status as the dependent variable and genotype as the covariate, in addition to adjustments for age and gender as additional covariates. ^¶^ Adjusted for the effect of covariates: age and gender. *P*_HWE_: *p* value for Hardy–Weinberg Equilibrium. [OR, Odds ratio; NS, Non-significant; CI, Confidence interval; SNP, Single nucleotide polymorphism].

**Table 4:** Output of the Cox regression model for rs17501292 and rs2067087 genotypes using groups as covariates.

	Estimates					
SNP	Genotypes	Beta	Standard Error	*p*-Value	HR (Beta)	95% C.I
rs17501292	GG	Ref.	Ref.	Ref.	1	Ref.
	TG	−2.25	0.338	<0.0001	0.105	0.054–0.205
	TT	−2.664	0.334	<0.0001	0.07	0.036–0.134
rs2067087	CC	Ref.	Ref.	Ref.	1	Ref.
	GC	−0.947	0.275	0.001	0.388	0.227–0.665
	GG	−0.786	0.473	NS	0.456	0.18–1.152

[C.I, confidence interval; HR, hazard ratio; NS, non-significant.].

**Table 5:** Association of HOTTIP gene SNPs with prognosis.

SNP	Genetic Model	Genotype	Favorable Prognosis (APRI Score < 0.5) (n = 83)	Unfavorable Prognosis (APRI Score ≥ 0.5) (n = 115)	X^2^	*p*-Value	OR (95%C.I)	*p*-Value ^¶^
			N (%)	N (%)				
rs17501292	Codominant	TT	54 (65.06%)	55 (47.83%)	6.396	0.041	0.343 (0.115–1.018)	0.044 ^¶^
		TG	24 (28.92%)	45 (39.13%)			0.635 (0.203–0.991)	
		GG	5 (6.02%)	15 (13.04%)			1 (Ref.)	
	Dominant	GG + TG vs. TT	29 (34.94%) vs. 54 (65.06%)	60 (52.17%) vs. 55 (47.83%)	5.786	0.02	0.491 (0.274–0.878) vs. 1 (Ref.)	0.016 ^¶^
	Recessive	TT + TG vs. GG	78 (93.98%) vs. 5 (6.02%)	100 (86.96%) vs. 15 (13.04%)	2.616	NS	0.428 (0.147–1.242) vs. 1 (Ref.)	NS ^¶^
	---	T allele vs. G allele	132 (79.52%) vs. 34 (20.48%)	155 (67.39%) vs. 75 (32.61%)	7.107	0.009	0.559 (0.347–0.901) vs. 1 (Ref.)	0.018 ^¶^
rs2067087	Codominant	GG	6 (7.23%)	12 (10.43%)	8.623	0.013	0.947 (0.319–2.81)	0.012 ^¶^
		GC	50 (60.24%)	45 (39.13%)			0.41 (0.222–0.757)	
		CC	27 (32.53%)	58 (50.43%)			1 (Ref.)	
	Recessive	CC + GC vs. GG	77 (92.77%) vs. 6 (7.23%)	103 (89.56%) vs. 12 (10.43%)	0.599	NS	1.518 (0.541–4.257) vs. 1 (Ref.)	NS ^¶^
	Dominant	GG + GC vs. CC	56 (67.47%) vs. 27 (32.53%)	57 (49.56%) vs. 58 (50.43%)	6.308	0.014	0.468 (0.26–0.844) vs. 1 (Ref.)	0.012 ^¶^
	---	G allele vs. C allele	62 (37.35%) vs. 104 (62.65%)	69 (30%) vs. 161 (70%)	2.352	NS	0.805 (0.522–1.242) vs. 1 (Ref.)	NS ^¶^

A χ2 test was performed for various genotypes, T allele, G allele, and C allele. OR was calculated via binary logistic regression with prognosis as the dependent variable and genotype as the covariate, in addition to adjusting for age and gender as additional covariates. ^¶^ Adjusted for the effect of covariates: age and gender. [NS, non-significant.].

**Table 6:** (A) Stratified analysis of the effect of rs17501292 (T/G) SNP on HCC metastasis risk by age and gender. (B). Stratified analysis of the effect of rs2067087 (G/C) SNP on HCC metastasis risk by age and gender.

**(A)**						
**rs17501292**		**TT**	**TG**	**GG**	***p*** **-value**	**OR (95% CI)**
**Parameter**		**Non-metastatic/Metastatic**				
Age	>60	55/16	28/19	4/5	NS	1 (Ref.)
	≤60	25/13	10/12	7/4		1.5 (0.81–2.807)
Gender	Male	54/23	25/25	5/6	0.045	1 (Ref.)
	Female	26/6	13/6	6/3		0.49 (0.244–0.983)
**(B)**						
**rs2067087**		**GG**	**GC**	**CC**	***p*** **-value**	**OR (95% CI)**
**Parameters**		**Non-metastatic/Metastatic**				
Age, n	>60	9/5	42/12	36/23	NS	1 (Ref.)
	≤60	2/2	30/11	10/16		1.796 (0.95–3.395)
Gender	Male	7/3	48/20	29/31	0.043	1 (Ref.)
	Female	4/4	24/3	17/8		0.487 (0.242–0.978)

The values are expressed as numbers (non-metastatic/metastatic HCC patients). [CI, confidence interval; OR, odds ratio; NS, non-significant].

**Table 7:** Haplotype and joint analysis of rs17501292 (T/G) and rs2067087 (G/C) polymorphisms in non-metastatic HCC patients compared with metastatic HCC patients.

Combined		Non-Metastatic, n (%)	Metastatic, n (%)	*p*-Value	OR (95% C.I)
Alleles	TG	84 (32.56%)	29 (21.01%)	Ref.	1 (Ref.)
	TC	114 (44.19%)	60 (43.48%)	NS	0.656 (0.388–1.109)
	GG	10 (3.88%)	8 (5.8%)	NS	0.432 (0.155–1.198)
	GC	50 (19.37%)	41 (29.71%)	0.004	0.421 (0.233–0.76)
Genotypes	TT + GG	9 (6.98%)	4 (5.8%)	Ref.	1 (Ref.)
	TT + GC	47 (36.43%)	11 (15.94%)	NS	1.899 (0.493–7.313)
	TT + CC	24 (18.61%)	14 (20.29%)	0.027	0.762 (0.198–2.938)
	GG + GG	0 (0%)	1 (1.45%)	NS	4.315 × 10^−9^ (4.315 × 10^−9^–4.315 × 10^−9^)
	GG + GC	8 (6.21%)	4 (5.8%)	1	0.889 (0.165–4.777)
	GG + CC	3 (2.31%)	4 (5.8%)	1	0.333 (0.05–2.239)
	TG + GG	2 (1.55%)	2 (2.9%)	NS	0.444 (0.045–4.374)
	TG + GC	17 (13.18%)	8 (11.59%)	NS	0.944 (0.222–4.014)
	TG + CC	19 (14.73%)	21 (30.43%)	0.002	0.402 (0.106–1.522)

The values are expressed in numbers. Non-metastatic HCC, n = 129; metastatic HCC, n = 69. [C.I, confidence interval; OR, odds ratio; NS, non-significant.].

**Table 8:** Univariate analysis to predict the risk of HCC metastasis using the examined SNPs.

SNP	Coefficient	SE	*p*-Value	OR (95% CI)	*p*-Value ^¶^	OR (95% CI) ^¶^
rs17501292						
(GG + TG vs. TT)	−0.684	0.338	0.043	0.505 (0.26–0.978)	0.035	0.482 (0.245–0.949)
(TT + TG vs. GG)	−0.223	0.529	0.673	0.8 (0.283–2.258)	NS	0.755 (0.259–2.2)
rs2067087						
(CC + GC vs. GG)	0.808	0.551	0.143	2.243 (0.762–6.603)	NS	2.807 (0.92–8.566)
(GG + GC vs. CC)	−0.904	0.334	0.007	0.405 (0.21–0.779)	0.004	0.374 (0.191–0.733)

Logistic regression analysis to predict the risk of HCC. *p* ^¶^ adjusted for age and gender by GLM. Non-metastatic HCC, n = 129; metastatic HCC, n = 69. [CI, confidence interval; HCC, hepatocellular carcinoma; OR, odds ratio; NS, non-significant; SNP, single-nucleotide polymorphism; SE, Standard error].

**Table 9:** (A). Probabilities and docking scores for the siRNA population against HOTTIP. (B). The 3D structure, energy, and hybridization energy of the top 2 chosen targets.

**(A)**			
**Target** **Position**	**HNADOCK SCORE**	**HDOCK SOCRE**	**Probability from catRAPID omics (Cutoff 0.5)**
4612–4634	−402.72	−576.82	0.5
4611–4633	−353.35	−594.53	0.5
4557–4579	−334.19	−544.77	0.5
4555–4577	−369.37	−496.08	0.5
4554–4576	−318.24	462.63	0.55
4493–4515	−263.47	−500.94	0.5
4488–4510	−462.63	−675.28	0.6
4486–4508	−280.45	−486.33	0.55
4349–4371	−404.71	−616.83	0.55
4333–4355	−340.61	−596.16	0.55
4332–4354	−386.17	−739.56	0.55
4330–4352	−368.11	−454.26	0.5
4321–4343	−490.99	−555.41	0.5
4296–4318	−389.62	−719.76	0.5
4274–4296	−424.95	−615.75	0.5
807–829	−383.09	−490.14	0.5
1329–1351	−314.26	−439.6	0.5
1333–1355	−373.5	−577.07	0.5
3133–3155	−266.42	−420.54	0.5
3145–3167	−366.66	−570.78	0.6
3593–3615	−318.37	−514.3	0.5
3601–3623	−350.06	−529.3	0.55
3604–3626	−282.18	−420.02	0.5
3616–3638	−368.18	−565.9	0.5
3793–3815	−427.73	−476.33	0.5
3801–3823	−334.59	−553.76	0.5
3802–3824	−308.59	−484.38	0.5
4014–4036	−336.61	−656.42	0.5
4049–4071	−408.25	−693.16	0.5
4053–4075	−444.49	−758.07	0.5
4160–4182	−392.12	−546.08	0.5
4162–4184	−444.79	−628.17	0.5
4168–4190	−370.1	−629.02	0.5
4169–4142	−470.91	−747.91	0.5
4205–4227	−350.03	−562.97	0.5
**(B)**			
**Interaction**	**3D**	**Energy (kcal/mol)**	**Hybridization energy**
4169–4142	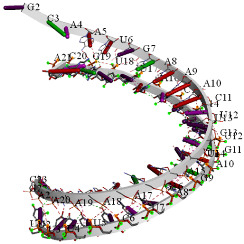	−29.4	−29.8
4321–4343	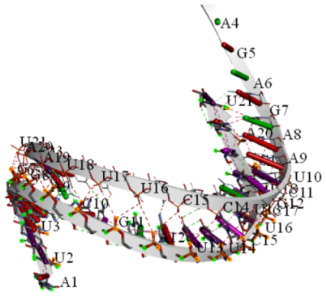	−30.04	−32.1

**Table 10:** Direct best siRNA results in inhibition of the binding site between HOTTIP and WDR5.

	Target Sequence	RNA Oligo Sequences	Functional siRNA Selection:	Seed-Duplex Stability	Specificity Check: Off-Target Hits # with Indicated Mismatches (Strand)	
Target position	21nt target + 2nt overhang	21nt guide (5′→3′) 21nt passenger (5′→3′)	Ui-Tei	(Tm);	Guide (+)	Passenger (-)
4321–4343	ATGAGAGAATCGTCCTTTAATAG	AUUAAAGGACGAUUCUCUCAU GAGAGAAUCGUCCUUUAAUAG	U	11.0 °C	19.1 °C	2 (https://sidirect2.rnai.jp/detail.cgi?seq=TGAGAGAATCGTCCTTTAA&strand=plus&spe=hs_refseq220, accessed on 7 June 2024)
4169–4191	TGCAATGAAACTCTAGAAAAAGC	UUUUUCUAGAGUUUCAUUGCA CAAUGAAACUCUAGAAAAAGC	U	7.1 °C	7.4 °C	2 (https://sidirect2.rnai.jp/detail.cgi?seq=GCAATGAAACTCTAGAAAA&strand=plus&spe=hs_refseq220, accessed on 7 June 2024)

[# = number].

## Data Availability

The original contributions presented in this study are included in the article/Supplementary Material. Further inquiries can be directed to the corresponding author(s).

## Supplementary Materials

The following supporting information can be downloaded at: https://www.mdpi.com/article/10.3390/ijms27052108/s1.

## Abbreviations

The following abbreviations are used in this manuscript:

AFP	Alpha-Fetoprotein
ALP	Alkaline Phosphatase
ALT	Alanine Transaminase
ANOVA	Analysis of Variance
APRI	AST to Platelet Ratio Index
AST	Aspartate Transaminase
BA	Basophils
BMI	Body Mass Index
CA19.9	Cancer Antigen 19.9
CBC	Complete Blood Count
CEA	Carcinoembryonic Antigen
CI	Confidence Interval
CRP	C-Reactive Protein
CT	Computed Tomography
D.Bil	Direct Bilirubin
DGE	Differential Gene Expression
DM	Diabetes Mellitus
EDTA	Ethylenediaminetetraacetic Acid
ES	Edmondson–Steiner
GGT	Gamma Glutamyl Transferase
GLM	General Linear Model
GO	Gene Ontology
H3K4	Histone H3 lysine 4
HBV	Hepatitis B Virus
HCC	Hepatocellular Carcinoma
HCT%	Hematocrit Percentage
HCV	Hepatitis C Virus
HGB	Hemoglobin
HOTTIP	HOXA Transcript at the distal Tip
HTN	Hypertension
INR	International Normalized Ratio
KEGG	Kyoto Encyclopedia of Genes and Genomes
LD	Linkage Disequilibrium
LIHC	Liver Hepatocellular Carcinoma
LncRNAs	Long non-coding RNAs
LNM	Lymph Node Metastasis
MAF	Minor Allele Frequency
MCH	Mean Corpuscular Hemoglobin
MCHC	Mean Corpuscular Hemoglobin Concentration
MCV	Mean Corpuscular Volume
MLL	Mixed Lineage Leukemia
MO	Monocytes
NCBI	National Center for Biotechnology Information
NS	Non-significant
OR	Odds Ratio
OS	Overall Survival
PC	Prothrombin Concentration
PCA	Principal Component Analysis
PCs	Principal Components
PET-CT	Positron Emission Tomography and Computed Tomography
PLT	Platelet Count
PPI	Protein- Protein Interaction
PT	Prothrombin Time
RBG	Random Blood Glucose
RDW%	Red Cell Distribution Width Percentage
REC	Research Ethics Committee
ROC	Receiver Operating Characteristic
RT	Room Temperature
RT-PCR	Real-Time Polymerase Chain Reaction
SE	Standard Error
S.E.M	Standard Error of Mean
siRNA	small interfering RNA
SNP	Single-Nucleotide Polymorphism
SPSS	Statistical Package for Social Sciences
T.Bil	Total Bilirubin
TC	Total Cholesterol
TG	Triglycerides
US	Ultrasound
VEP	Variant Effect Predictor
WBCs	White Blood Cells
WMA	World Medical Association
WDR5	WD Repeat Domain 5
YRIL	Yoruba in Ibadan, Nigeria
