# Supplementary material for: HOTTIP-Variants in Liver Cancer Metastasis Prognosis: A Clinical Study with Bioinformatics and siRNAs Targeting HOTTIP-WDR5 via Molecular Docking, a Step-Toward ncRNA Precision
Source: Int J Mol Sci. 2026 Feb 24;27(5):2108. doi: 10.3390/ijms27052108 (PMC12984522; doi:10.3390/ijms27052108)
Supplement: Supplementary file 1 [file ijms-27-02108-s001.zip › Supplementary Figure S1.pdf]

Article

HOTTIP-Variants in Liver Cancer Metastasis Prognosis: A Clinical Study with Bioinformatics and siRNAs Targeting HOTTIP-WDR5 via Molecular Docking, a Step Toward ncRNA Precision

Mona G. El-Sisi [1], Sara M. Radwan [1], Sameh S. Ali [2], Mohamed Y. Mostafa [3] and Nadia M. Hamdy [1,*]

[1] Department of Biochemistry and Molecular Biology, Faculty of Pharmacy, Ain Shams University, Cairo 11566, Egypt
[2] Research Department, Children's Cancer Hospital Egypt, Cairo 57357, Egypt
[3] Department of Clinical Oncology, Faculty of Medicine, Ain Shams University, Cairo 11566, Egypt
* Correspondence: nadia_hamdy@pharma.asu.edu.eg

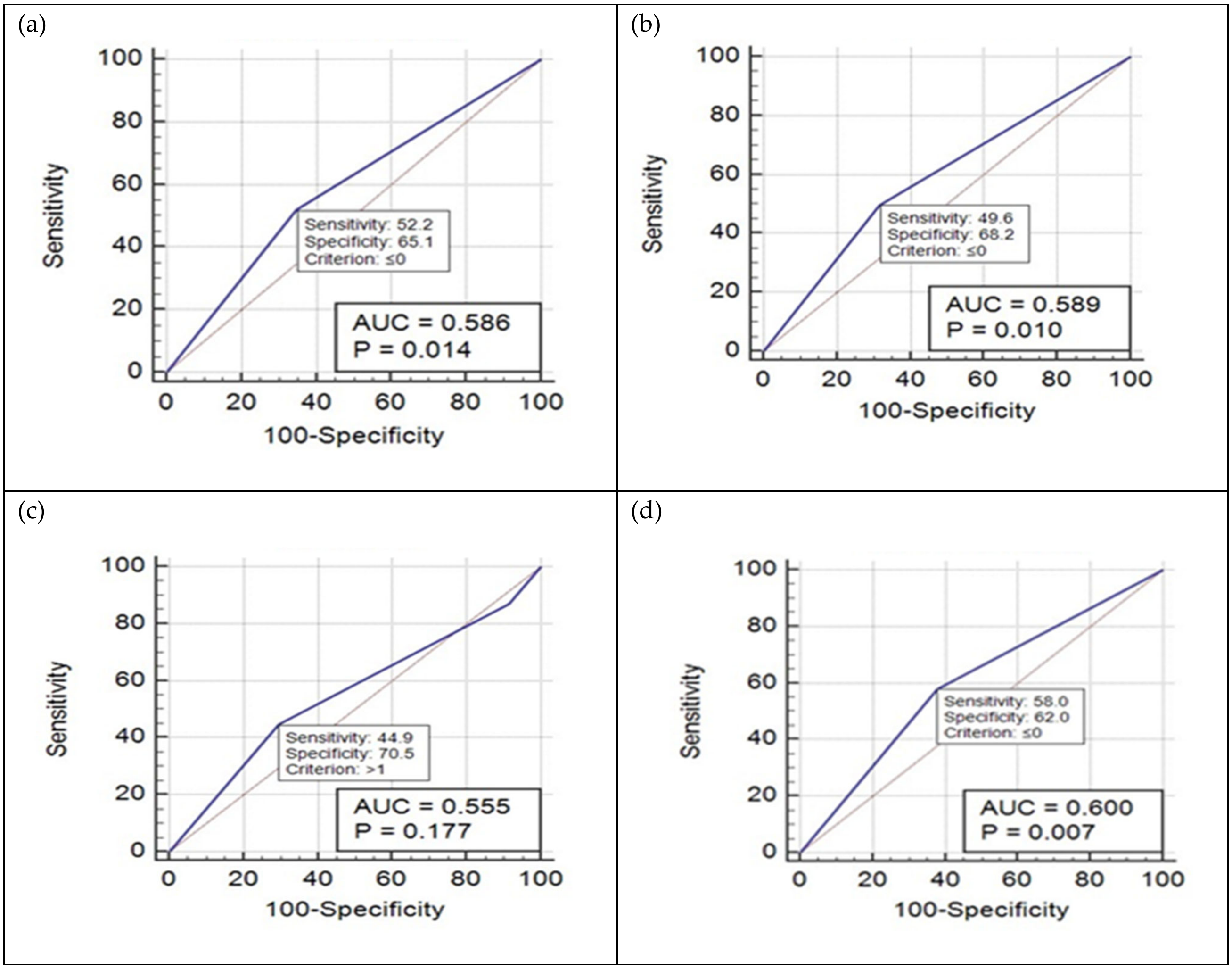

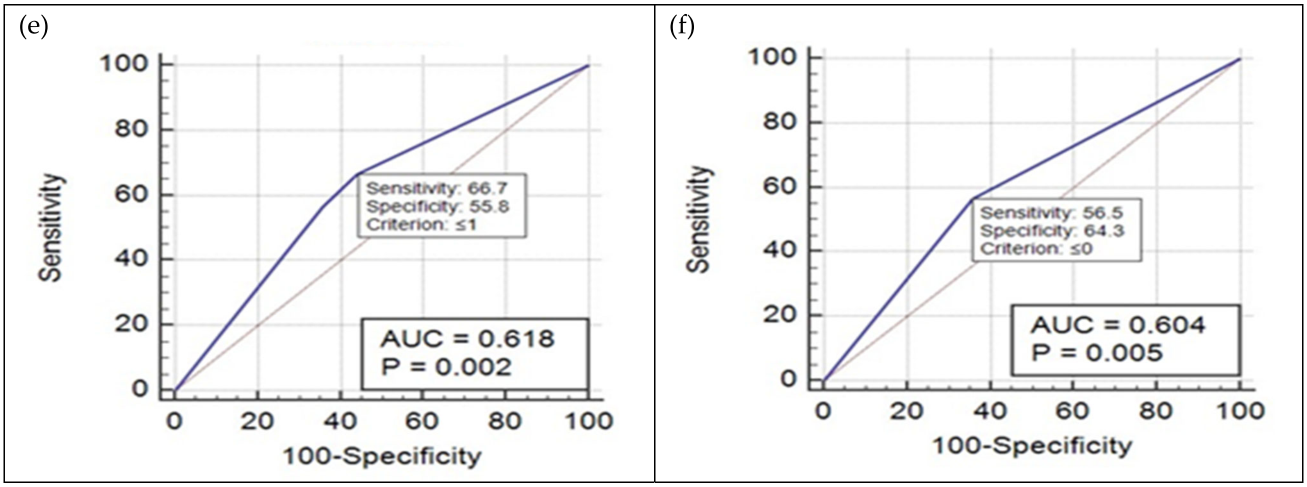

Supplementary Figure S1. ROC curves for evaluating (a) the prognostic value of rs17501292 codominant model (*GG* vs. *TG* vs. *TT*). (b) the prognostic value of rs2067087 dominant model (*GG* + *GC* vs. *CC*), (c) the diagnostic value of rs17501292 codominant model (*GG* vs. *TG* vs. *TT*). (d) the diagnostic value of rs17501292 dominant model (*GG* + *TG* vs. *TT*). (e) the diagnostic value of rs2067087 codominant model (*GG* vs. *GC* vs. *CC*). (f) the diagnostic value of rs2067087 dominant model (*GG* + *GC* vs. *CC*). [AUC, Area under the curve].
